# Identification of allosteric inhibitors of the ecto-5'-nucleotidase (CD73) targeting the dimer interface

**DOI:** 10.1371/journal.pcbi.1005943

**Published:** 2018-01-29

**Authors:** Rahila Rahimova, Simon Fontanel, Corinne Lionne, Lars Peter Jordheim, Suzanne Peyrottes, Laurent Chaloin

**Affiliations:** 1 Institut de Recherche en Infectiologie de Montpellier (IRIM), Univ. Montpellier, CNRS, Montpellier, France; Institut de Biologie Computationnelle (IBC), Montpellier, France; 2 Institut de Génomique Fonctionnelle (IGF), Univ. Montpellier, CNRS, Montpellier, France; 3 Centre de Biologie Structurale (CBS), Univ. Montpellier, CNRS, INSERM, Montpellier, France; 4 Centre Léon Bérard (CLB), Centre de Recherche en Cancérologie de Lyon (CRCL), Univ. de Lyon, INSERM, CNRS, Lyon, France; 5 Institut des Biomolécules Max-Mousseron (IBMM), CNRS, Univ. Montpellier, ENSCM, Montpellier, France; Icahn School of Medicine at Mount Sinai, UNITED STATES

## Abstract

The ecto-5’-nucleotidase CD73 plays an important role in the production of immune-suppressive adenosine in tumor micro-environment, and has become a validated drug target in oncology. Indeed, the anticancer immune response involves extracellular ATP to block cell proliferation through T-cell activation. However, in the tumor micro-environment, two extracellular membrane-bound enzymes (CD39 and CD73) are overexpressed and hydrolyze efficiently ATP into AMP then further into immune-suppressive adenosine. To circumvent the impact of CD73-generated adenosine, we applied an original bioinformatics approach to identify new allosteric inhibitors targeting the dimerization interface of CD73, which should impair the large dynamic motions required for its enzymatic function. Several hit compounds issued from virtual screening campaigns showed a potent inhibition of recombinant CD73 with inhibition constants in the low micromolar range and exhibited a non-competitive inhibition mode. The structure-activity relationships studies indicated that several amino acid residues (D366, H456, K471, Y484 and E543 for polar interactions and G453-454, I455, H456, L475, V542 and G544 for hydrophobic contacts) located at the dimerization interface are involved in the tight binding of hit compounds and likely contributed for their inhibitory activity. Overall, the gathered information will guide the upcoming lead optimization phase that may lead to potent and selective CD73 inhibitors, able to restore the anticancer immune response.

## Author summary

Nucleotidases play a central role in maintaining the nucleotide pool homeostasis and the only extracellular member of this family, CD73, has become an attractive target in oncology because of its high expression level on immune and cancer cells. In the tumor microenvironment, CD73-generated adenosine prevents the pro-inflammatory response and is considered as a potent immune suppressor. The current study aimed at developing new CD73 inhibitors by targeting an allosteric binding site in order to block the enzyme dynamics and therefore its enzymatic function. Most of the existing inhibitors have been elaborated on the basis of the substrate skeleton and act as competitive inhibitors. Here, four non-competitive compounds are presented with an inhibition constant in the low micromolar range. This study confirms the existence of an allosteric binding site located at the dimerization interface allowing modulation of the enzyme activity by small molecules, similarly to a previously described monoclonal antibody.

## Introduction

The immune response constitutes a major barrier for preventing cancer progression through the activation of T cells and subsequent release of pro-inflammatory cytokines. This process is initiated and tightly regulated by extracellular ATP which impacts a large variety of cells (T and B lymphocytes, NK, macrophages, DC, neutrophils and vascular endothelial cells) through the binding to P2X and P2Y receptors, inducing persistent inflammation and regulatory cell inhibition [1–3]. In healthy tissues, the extracellular ATP concentration is very low and estimated between 10 and 100 nM whereas in solid tumors, ATP is abundantly released in particular by dying cells, and through secretion, and its concentration can reach a few hundreds of micromolar [4]. In the tumor microenvironment, ATP usually acts as an alarm signal allowing the recruitment of immune cells and contributing to the immunogenic cell death process. However, when high ATP concentrations are associated with a high expression level of CD39 and CD73 on both immune and cancer cells, ATP is rapidly and successively degraded into AMP and then adenosine by the concerted activities of these two ectonucleotidases [5]. As a consequence, an abnormal adenosine concentration is produced in the tumor microenvironment and induces a potent suppression of the antitumor immune response through the adenosine binding to P1 receptors (mainly A2a and A2b) expressed on immune cells [6–9].

Ecto-5’-nucleotidase, or CD73 (EC 3.1.3.5), is a glycosylphosphatidylinositol (GPI) anchored cell surface protein that is expressed as a non-covalently linked homodimer on endothelial, immune and tumor cells. CD73 also exists as a soluble and circulating form with similar enzymatic activity to its membrane-attached form. Intriguingly, this soluble form was also found in cell and organ crude extracts probably generated by a phospholipase activity on the GPI-anchored precursor. However, the precise role of this intracellular form is not fully understood in particular because of the high intracellular ATP concentration making the enzyme inactive [10]. In human peripheral blood, CD73 is expressed on most of B lymphocytes, T cells including Th17, NK and myeloid-derived suppressor cells [3]. These cells can also co-express CD39 and CD73 [11]. In the tumor microenvironment in which hypoxia is predominant, CD73 has been shown to be overexpressed in various types of solid tumors as well as endothelial cells [12]. This encompasses several cancers such as colorectal, breast, bladder, pancreas, ovarian, leukemia and melanoma, as recently reviewed in [13], and is generally associated with poor prognosis in patients receiving anticancer treatments [14]. Few exceptions have been described pointing out CD73 as good prognosis marker as for the clinical study of endometrial and breast carcinomas [15,16]. This discrepancy between opposed roles of CD73 may be due to specific changes in endometrial cancers (endometrial epithelial barrier integrity) or may be a consequence of predominant presence of the soluble form of CD73 (sCD73). Indeed, higher plasma concentrations of sCD73 were determined in cancer patients or patients suffering of acute inflammatory pancreatitis compared to healthy individuals [17,18]. These studies suggest that the upregulation of sCD73 levels in blood may be a prognosis marker of tissue inflammation and tumor hypoxia. Moreover, CD73 overexpression has been shown to promote cell proliferation, migration, invasion and attachment to the extracellular matrix in human breast cancer [19,20] through the action of adenosine binding to A1 and A3 receptors [21]. CD73 deficiency was also studied in mice and correlated with resistance to *in vivo* development of carcinoma [22] or with antitumor immunity improvement [23]. The role of CD73-produced adenosine in cancer progression and metastasis has been evidenced by the use of either monoclonal antibodies [24] or siRNA [25] blocking CD73 enzyme activity. As a consequence, the immune response through ATP purinergic signaling could be restored. For all these reasons, CD73 has been considered as a promising therapeutic target to develop new anticancer therapies. The first described CD73 inhibitors were ADP, ATP and adenosine 5’-[α,β-methylene]diphosphate (a non-hydrolysable ADP analog named APCP), all acting as competitive inhibitors [10]. Subsequently, small molecule inhibitors derived from APCP have been recently designed and studied showing potent competitive inhibition of CD73 [26]. However, competitive inhibitors, especially those targeting kinases, present several drawbacks such as low selectivity profiles [27,28] or weak efficiency when competing with high substrate concentrations. In order to overcome this problem, an alternative approach consists in the development of non-competitive or allosteric inhibitors interacting with the target outside the substrate binding site. As an evidence that occurred likely by chance, a new monoclonal antibody developed by MedImmune (MEDI9447), was shown to inhibit CD73 enzymatic activity through such a dual mechanism [29] involving a non-competitive inhibition. Although this approach was quite different in regard to small drug molecules, it demonstrates the proof of feasibility consisting in blocking the enzyme conformation and leading to CD73 inhibition. Interestingly, MEDI9447 did not compete with AMP and the antibody was able to prevent the conformational transition required for forming the enzyme active site. As illustrated by the crystal structures of this enzyme solved in the open and in the closed conformations [30,31], large dynamics domain motions are obviously required to form the closed active conformation (for both monomers). The di-metallic center is present in the *N-*domain while the adenosine moiety of the substrate binds to the *C-*domain and AMP hydrolysis will occur only after a large closure motion mediated by a rotation of the N-domain of up to 114° [30]. The objective of the current study was to reproduce the dynamics of the enzyme in order to identify druggable cavities and small molecule inhibitors able to block the dynamics and thereby the associated enzymatic function.

To achieve this objective, we describe a bioinformatics approach that allowed the identification of new allosteric inhibitors (designated hereafter as “**RR**” compounds) targeting human CD73 and able to block efficiently its enzymatic function in the low micromolar range through a non-competitive inhibition mechanism. This is the first stage of a future drug development process and the selected lead compounds will require further structural optimization to envisage forthcoming *in vivo* applications. The final objective behind our search of new inhibitors is to restore the antitumor immune response by downregulating the extracellular adenosine concentration either by using **RR** compounds alone or in combination with immunotherapies.

## Results

### Enzyme conformational changes and cavity selection

**Fig 1:**
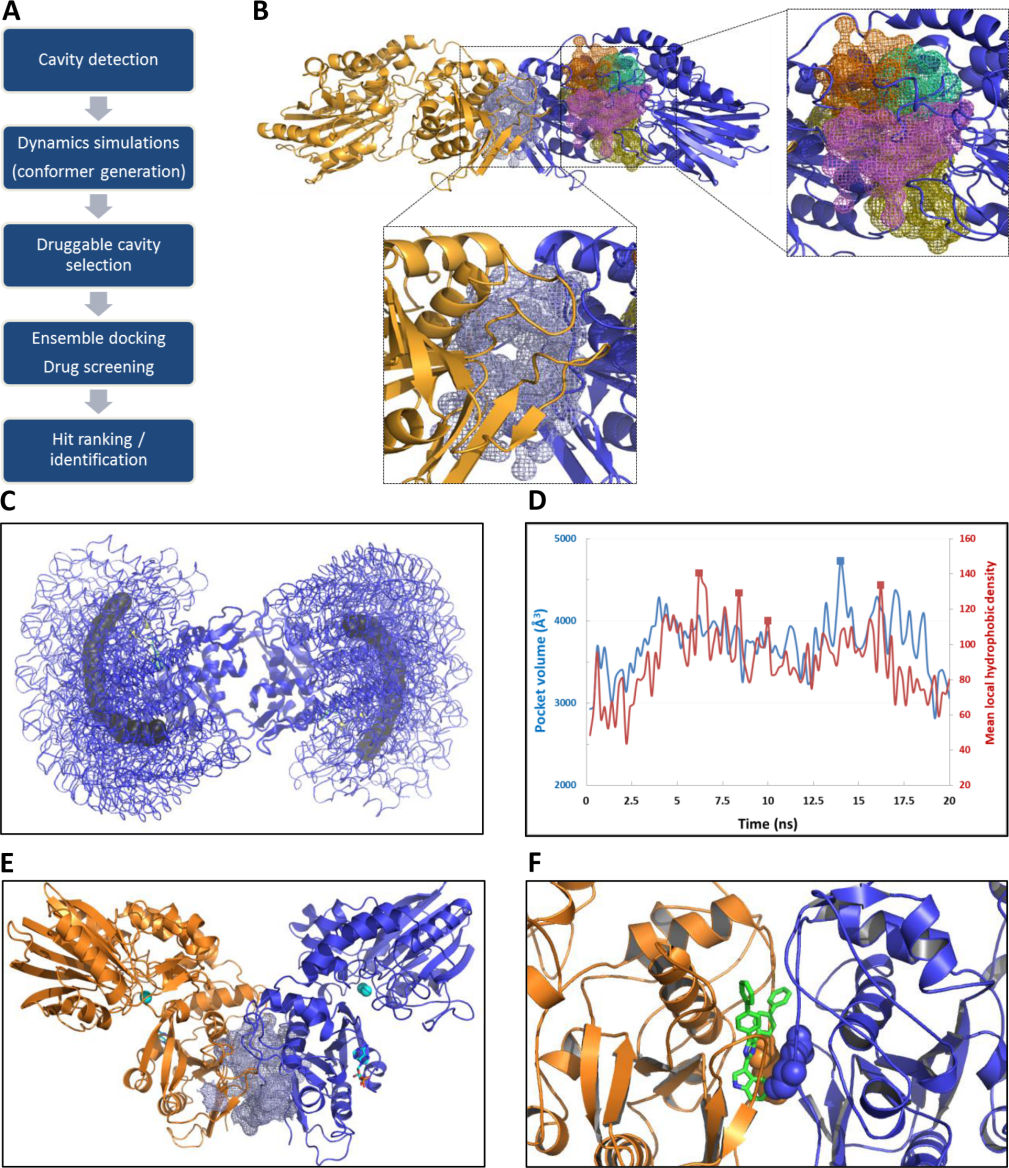
Structure-based drug design including cavity selection and dynamics of the enzyme target. (A) Flowchart illustrating the global strategy for developing allosteric CD73 inhibitors. (B) Five cavities detected using Fpocket on the closed dimeric form of CD73 (4H2G) and shown in colored mesh representations. (C) Top view of superimposed structures of CD73 during the TMD simulation highlighting the large rotating motion of N-domains (centers of mass depicted as spheres in arc shape). (D) Volumes changes and mean local hydrophobic densities observed during TMD for the blue cavity from panel “B” located at the dimerization interface. (E) Target cavity (mesh representation) outside the substrate binding site (AMP and Zn ions are depicted in cyan sticks). (F) Illustration of the target binding site in complex with one hit compound (green sticks) obtained by docking (Glu543 residues are depicted as spheres).

The overall strategy followed in this study for cavity selection and hit identification is schematically illustrated in Fig 1A. First by analyzing the crystal structure (4H2G) and by using the Fpocket program, we detected five potential druggable cavities (Fig 1B). For the selection of the most suitable cavity, these pockets must fulfill important criteria: i) a cavity located far away from the substrate binding site (to avoid competitive inhibition), ii) a cavity with a sufficient volume to afford the binding of drug-like molecules, iii) a cavity showing variation in size and volume during the dynamics (the final goal being to block dynamic motions of the enzyme), and iv) displaying a high mean local hydrophobic density as previously described for druggability [32]. Therefore, to evaluate their change in size and volume during the reaction, we performed molecular dynamics simulations enabling the selection of the most suitable and druggable cavity for virtual screening. Hence, TMD simulations were carried out to reproduce the large domain motions occurring during the reaction in both directions (from open to closed states and vice-versa). Indeed, to block the enzyme function, both directions are relevant as soon as the dynamic can be altered. We first focused on the closing direction in presence of the preferred substrate (AMP). Rigid body motions of the *N*-terminal domain toward the *C*-domain were observed with a preponderant rotating motion during closure, all together leading to the formation of the active site (Fig 1C and S1 Movie). Starting by an initial translational motion, both *N*-domains (residues ranging from 27 to 337) operate an anti-symmetric rotation around a central node formed by four amino acids (_335_STQE_338_) located between α-helix I and β-sheet 15 [30] (as shown in Fig 1C with the displacement of the center of mass of each *N*-domain). Similar results were obtained in the opposite direction except that large collective motions of the *N*-domain were slower at the beginning of the simulation (using the same force constant applied in both TMD simulations). This can be easily explained by the presence of the substrate that is tightly bound to the *C*-domain through strong electrostatic interactions between the phosphate oxygen atoms and the two zinc ions. Using 100 conformers issued from the simulations (Fig 1D), a cavity located at the junction of both *C*-domains (called hereafter, dimerization interface, Fig 1E) was the only pocket meeting all the druggability criteria previously defined, and was therefore selected as target binding site for virtual screening. As shown in Fig 1D, results obtained by MDpocket analyses indicated that the volume of this cavity is rather large and highly fluctuating (from 2815 to 4732 Å^3^) and also that the mean local hydrophobic density was increasing for several conformers (delineated by square symbols in Fig 1D). Therefore, five representative conformers (denoted as C1 to C5, S1 Fig) were selected for an ensemble docking to mimic both the dynamics of the enzyme (volume of the cavity) and druggability according to the apolar descriptor. The center of the screening area was defined by both Glu543 residues located in this interface as shown in Fig 1F in the presence of one hit compound. The initial and final states of the simulation which correspond to the experimental crystal structures were not included for virtual screening as the previous defined criteria were not fully satisfied (smaller volume of the pocket and apolar contribution). However, the overall structural quality of the selected conformers issued from the TMD simulation was assessed by means of three different methods. First, RMSD of backbone atoms from both *C*-domains were computed using as reference structure either the open or the closed crystal structure (S1 Fig). Then, the Z-score (Prosa protein analysis tool) was calculated for each conformer and found to be very close to the ones computed for the experimental structures (S2 Fig). In addition, the Ramachandran diagrams (S3 Fig) were computed for all structures and indicated a very low violation rate. Indeed, about 1% of residues were found in outlier regions and these latter were located near the substrate binding site. Altogether, these results indicated that the conformers selected for the virtual screening display an overall excellent structural quality.

### Hit identification

**Fig 2:**
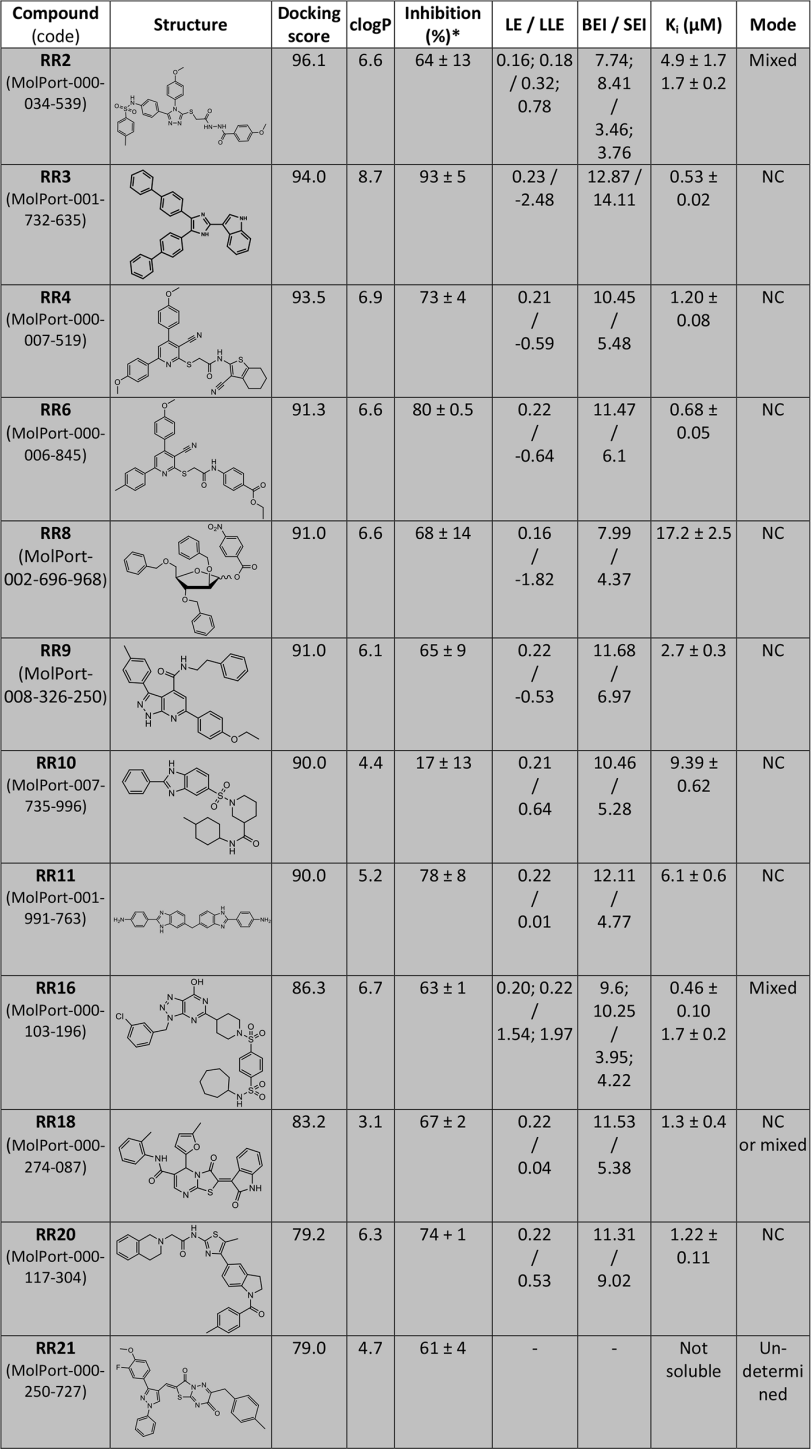
Selected hit compounds identified by docking at the dimerization interface of CD73 (full list in S1 Table). MolPort code and chemical structure of hit compounds ranked by docking score. In addition to clog *P* value, different metrics for ligand efficiency were computed: LE, LLE, BEI and SEI (see Materials and Methods section for details). Enzymatic inhibition by **RR** compounds (at 5 μM) performed with the purified recombinant CD73 Inhibition constants (K_i_) and mode are indicated (NC for non-competitive). *means +/- SD of three independent experiments.

Virtual screening of 324,400 compounds was carried out by targeting the dimerization interface on the five conformers issued from TMD simulation (ensemble docking). One could remark that half of the library was composed of compounds violating the Lipinski’s rules of 5 (either by a molecular weight > 500 or by a clog *P* > 5, or both). This feature was chosen on purpose for targeting the CD73 dimerization area as protein-protein interfaces are known to be highly apolar in comparison to exposed protein surfaces. The best hit compounds were selected from the top-ranked compounds obtained by AutoDock Vina and further rescored with Gold on each individual conformer. The final ranking was computed by averaging the score obtained with each conformer by Gold and a final round of selection was carried out to increase the structural diversity of hit compounds (Fig 2 and S1 Table for the full list of selected hit compounds denoted as **RR** and ranked by docking score). A cut-off value of the computed docking score was arbitrary selected at 70 leading to a docking score range between 96 and 71 (**RR1** to **RR28**). Additionally, five compounds (fragment-like compounds) from the initial library were kept for further testing because of their structural similarity with the best hit compounds (five last molecules listed in S1 Table). Most of the hit compounds showed high clog *P* indicating that the targeted dimerization interface has indeed a large hydrophobic area. The 33 best-ranked compounds did not share a common chemical structure but present some interesting features like a 3-D shape exploiting the chemical space by combining rigid scaffolds such as five- or six membered aromatic rings either as a tri-branched based molecule often encountered as for compounds **RR1-4**, **6**, **9**, **14**, **17–18**, **21** and **26**, or under an extended structure (**RR10-13**, **16**, **20**, **23**–**25, 27** and **28**). Interestingly, four compounds, **RR11**, **RR13**, **RR19** and **RR28** are dimeric structures composed of two identical components linked together (Fig 2 and S1 Table). This structural feature may be the indication of a common binding mode for each part of the compound with each monomer of the enzyme. Two molecules also exhibit a complex spatial organization as they include a ribose or a nucleoside scaffold bearing two or four aromatic protecting groups (**RR7** and **RR8**, respectively). The selection of this type of multi-branched structure may arise from the large surface to be occupied in the binding site of the enzyme and consequently, this feature may contribute to the blockade of the protein dynamics or activity. In addition, two highly similar structures were both selected from the screening process, compounds **RR4** and **RR6** with comparable inhibitory activities. The tri-branched core is almost identical (and based on a tetra-substituted pyridine ring) except the nature of the substituent on the side chain located in position 2 and either corresponding to the 2-amino-4,5,6,7-tetrahydro-benzo(b)thiophene (**RR4**) or to the ethyl 4-aminobenzoate (**RR6**). The end of the list shows some compounds with low scores, due to their low molecular weight comparable to fragments. These molecules were included for comparison and components analysis of larger molecules.

### Hit validation

**Fig 3:**
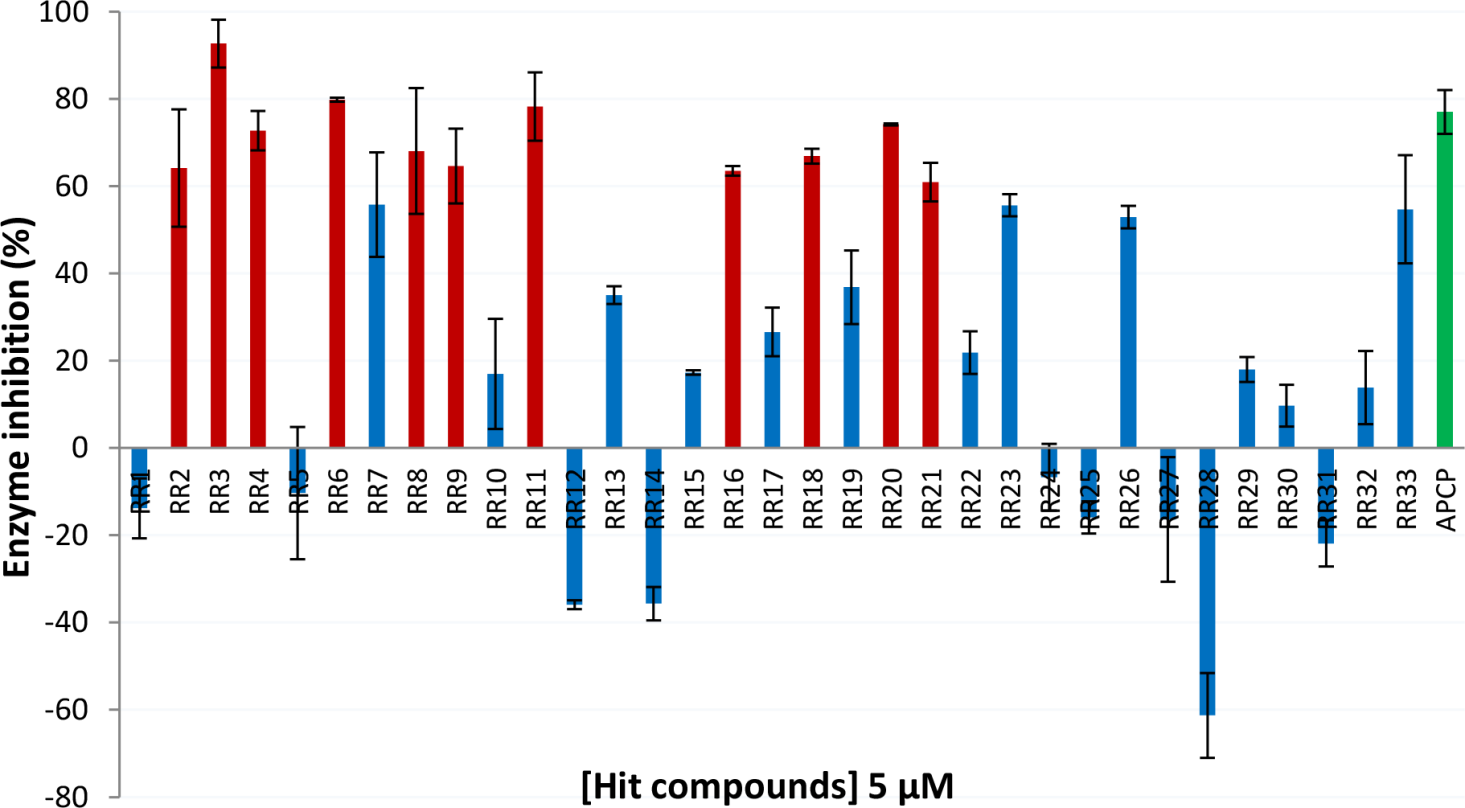
Enzymatic inhibition assay in the presence of RR compounds using the purified recombinant enzyme. Red bars indicate the most active **RR** compounds promoting an enzyme inhibition as efficiently as APCP (5 μM) used as a positive control (green bar). Values of inhibitions are means from three independent experiments ± SD and negative values reflect enzyme activation.

We tested the 33 best-ranked compounds for their potential inhibition of CD73 activity using the recombinant purified enzyme (S1 Table). For this purpose, the human dimeric soluble form of CD73 was expressed in insect cells using a pFastBac system to guaranty the presence of post-translational modifications since four potential glycosylation sites have been suggested [31]. Catalytic (*k*_cat_) and Michaelis (*K*_M_) constants were determined for the purified enzyme at 70.6 ± 2.4 s^-1^ and 4.8 ± 0.6 μM, respectively, leading to a catalytic efficiency of 14.7 μM^-1^.s^-1^. The enzyme was found three fold less active than the recombinant protein expressed in HEK cells [31], but the activity was comparable to the commercially available human enzyme, also produced in HEK. As shown in Fig 3, several compounds significantly inhibited the enzyme activity at a concentration as low as 5 μM. The most active ones, in terms of inhibition of CD73 enzyme activity, were **RR2-4, 6, 8–9, 11, 16, 18** and **20–21** which promoted an enzyme inhibition with a similar efficacy to that observed with APCP (Fig 3).

**Fig 4:**
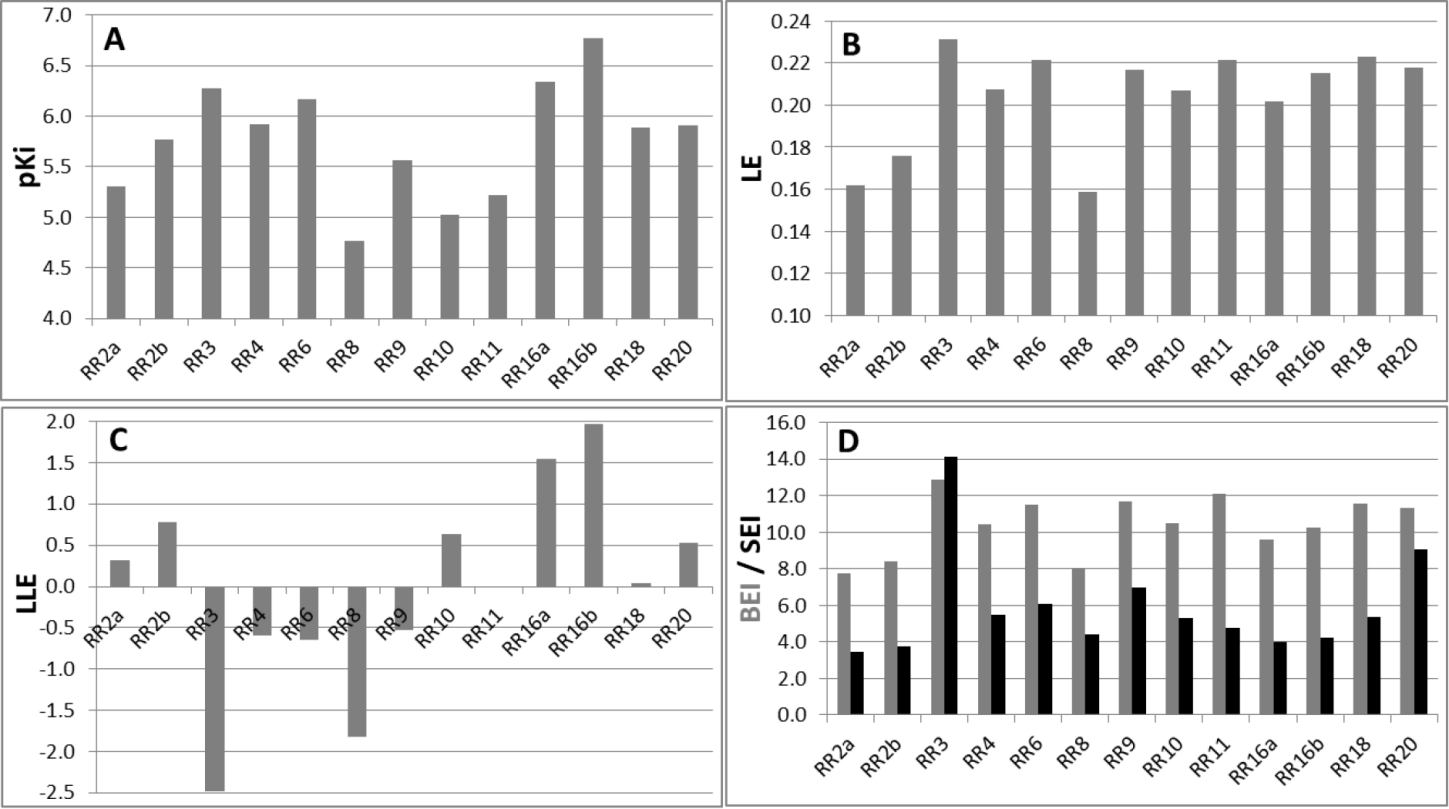
Comparison of hit compounds by using conventional metrics used in drug design. Inhibition constants (*K*_i_) are expressed as pK_i_ (A) and ligand (B), ligand-lipophilicity (C), binding and surface (D) efficiencies correspond to LE, LLE, BEI and SEI, respectively. Note that for compounds exhibiting a mixed inhibition mode, two inhibition constants (“a” and “b”) were determined as for **RR2** and **RR16**.

Higher concentrations of **RR** compounds (up to 200 μM) were tested giving a similar inhibition profile with larger standard errors due to the poor water-solubility of these compounds. Interestingly, some compounds gave negative values of inhibition meaning that they were able to activate the enzyme. This result was not surprising since allosteric compounds may play the opposite role by stabilizing a preferential conformation leading to higher enzymatic efficiency (positive allosteric regulators). **RR28** was the remarkable example of this type of enzyme enhancers and also **RR12** and **RR14** in a lesser extent. The strongest inhibitor was compound **RR3** that induced 93% of enzyme inhibition at a concentration of 5 μM. It was also predicted as the less water-soluble compound (clog *P* value: 8.7). Since these compounds were highly hydrophobic, we computed several metrics commonly used in drug design such as LE (ligand efficiency), LLE (ligand-lipophilicity efficiency), BEI (binding efficiency index) and SEI (surface-binding efficiency) (Fig 4 and S1 Table) in order to better evaluate the physicochemical properties that are preponderant in the binding efficiency [33,34]. LE is a simple but important indicator to select compounds according to their efficacy in respect to their atom number count. For orally available active compounds compliant with the rule of five, LE value should be at least 0.3 and this value is used for the selection of leads and needs to be maintained during the optimization process [35]. Here, all hit compounds exhibited low values of LE between 0.16 and 0.23 kcal.mol^-1^.HA^-1^ with **RR3** as best lead (LE = 0.23 with pKi = 6.28) followed by **RR6**, **9**, **11**, **16**, **18** and **20** (LE = 0.22). In contrast, LLE, which takes into account lipophilicity, indicated that the lipophilic contribution for **RR3** was not optimal (LLE = -2.48) and in this respect, **RR16** appeared as the better compound (LLE = 1.97 and LE = 0.22 with pKi = 6.77) and finally **RR4** and **RR6** with a moderate lipophilic contribution. Therefore, BEI and SEI were also computed to better appreciate which compound involves its molecular structure in the binding or the inhibition efficiency. While BEI takes into account only the molecular weight (global size), SEI encompasses the polar surface area (between 44 and 160 Å^2^), reflecting much better the occupation efficiency of the molecular surface. Here, **RR3** showed the highest value followed by **RR20**. Because of the structural shape analogy between **RR10** (weak inhibitor) and **RR16** (strong inhibitor), the kinetic inhibition assay was also carried out for **RR10**. Indeed, the determined *K*_i_ value (9.4 μM) was higher than the respective ones for **RR16** (0.46 and 1.7 μM, mixed inhibition). Although the inhibition mode was different for both compounds, the calculated LLE value was much higher for **RR16** than for **RR10** but these compounds showed very similar BEI or SEI values. According to these indexes, we can conclude that: **RR3** should be improved for a better use of its lipophilicity (LLE too small) as for **RR20**; **RR8** has very low pKi, LE and LLE values render difficult its optimization and finally, **RR16** could be improved for a better use of its molecular surface. Due to the low water solubility, this analysis could not be performed for compound **RR21**.

### Inhibition mode of the most active RR compounds

**Fig 5:**
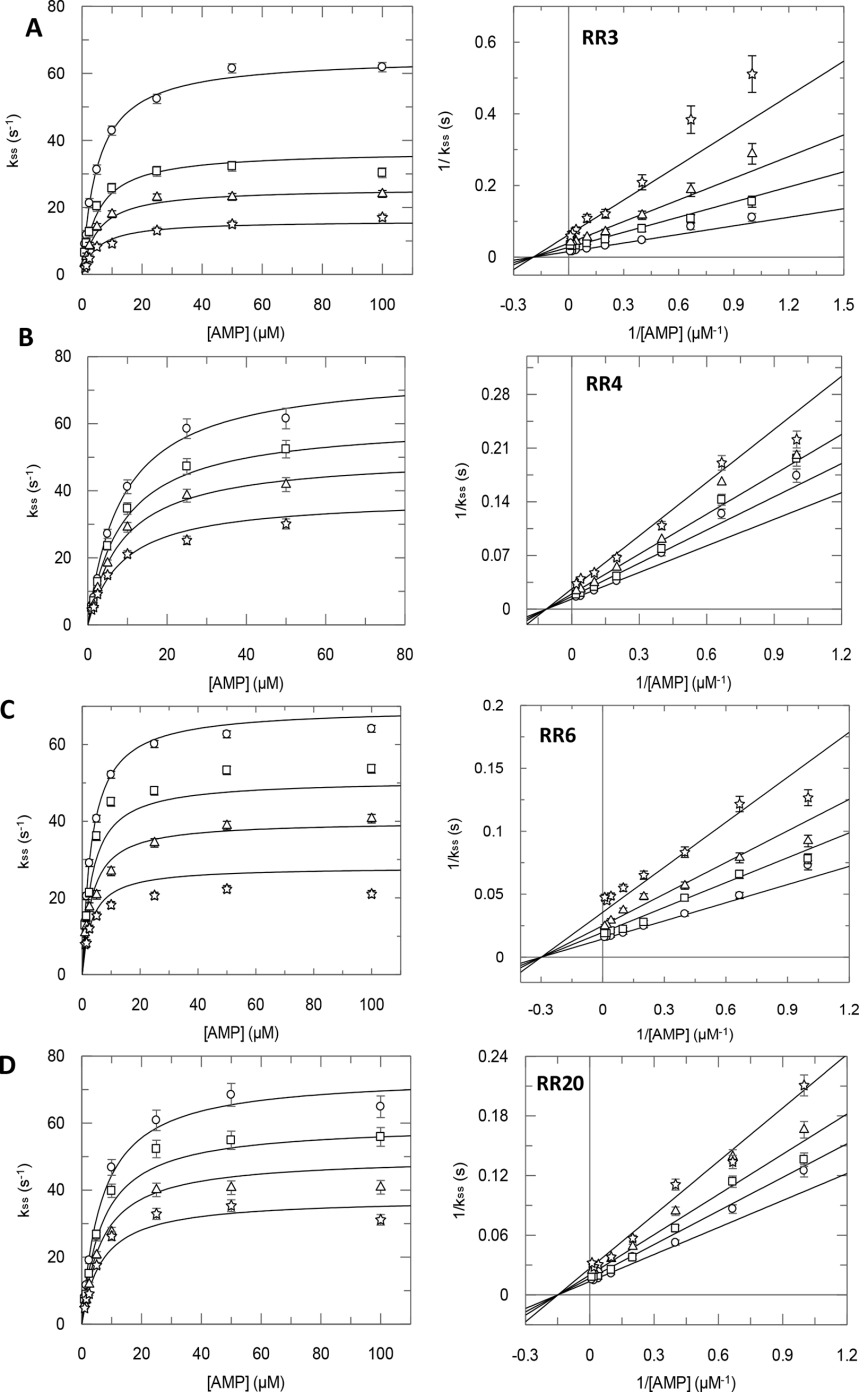
Determination of the kinetics inhibition profiles for the most representative compounds. Secondary plots and double-reciprocal of steady state rate constants as a function of AMP concentration in the absence (circles) or with increasing concentrations of hit compounds (squares, triangles and stars). (A): **RR3** at 0, 0.4, 0.8 and 1.6 μM; (B): **RR4** at 0, 0.3, 0.6 and 1.2 μM; (C): **RR6** at 0, 0.25, 0.5 and 1.0 μM; (D): **RR20** at 0, 0.3, 0.6 and 1.2 μM.

According to the location of the target binding site that was far away from the substrate binding site, **RR** compounds should impair the enzymatic function through a non-competitive inhibition mode. As expected, the kinetic mechanism describing the inhibitory activity for eight compounds (**RR3, 4, 6, 8–11** and **20**) was the non-competitive mode (Fig 5 and S1 Table). Nevertheless, for some compounds like **RR2** and **RR16**, a mixed inhibition mode was determined, indicating that they may also bind to the substrate binding site or to the enzyme-substrate complex. The inhibition profile could not be determined for poorly water-soluble compounds like **RR21**. Also, the inhibition mechanism could not be defined unambiguously for compound **RR18**, for which the experimental data fitted well with both mixed and non-competitive equations. The most active non-competitive inhibitors were **RR3** and **RR6** with *K*_i_ values of 0.52 ± 0.20 μM and 0.68 ± 0.05 μM, respectively (Fig 5A and 5C). **RR4** and **RR20** were less potent than the previous ones but still able to induce a strong inhibition of CD73 activity and exhibited a *K*_i_ around 1.2 μM (Fig 5B and 5D). **RR16** was deduced as a potent mixed inhibitor of CD73 with *K*_i_ and *K*_i_’ values of 0.46 ± 0.10 and 1.70 ± 0.20 μM, respectively.

### Structure-activity relationship studies

**Fig 6:**
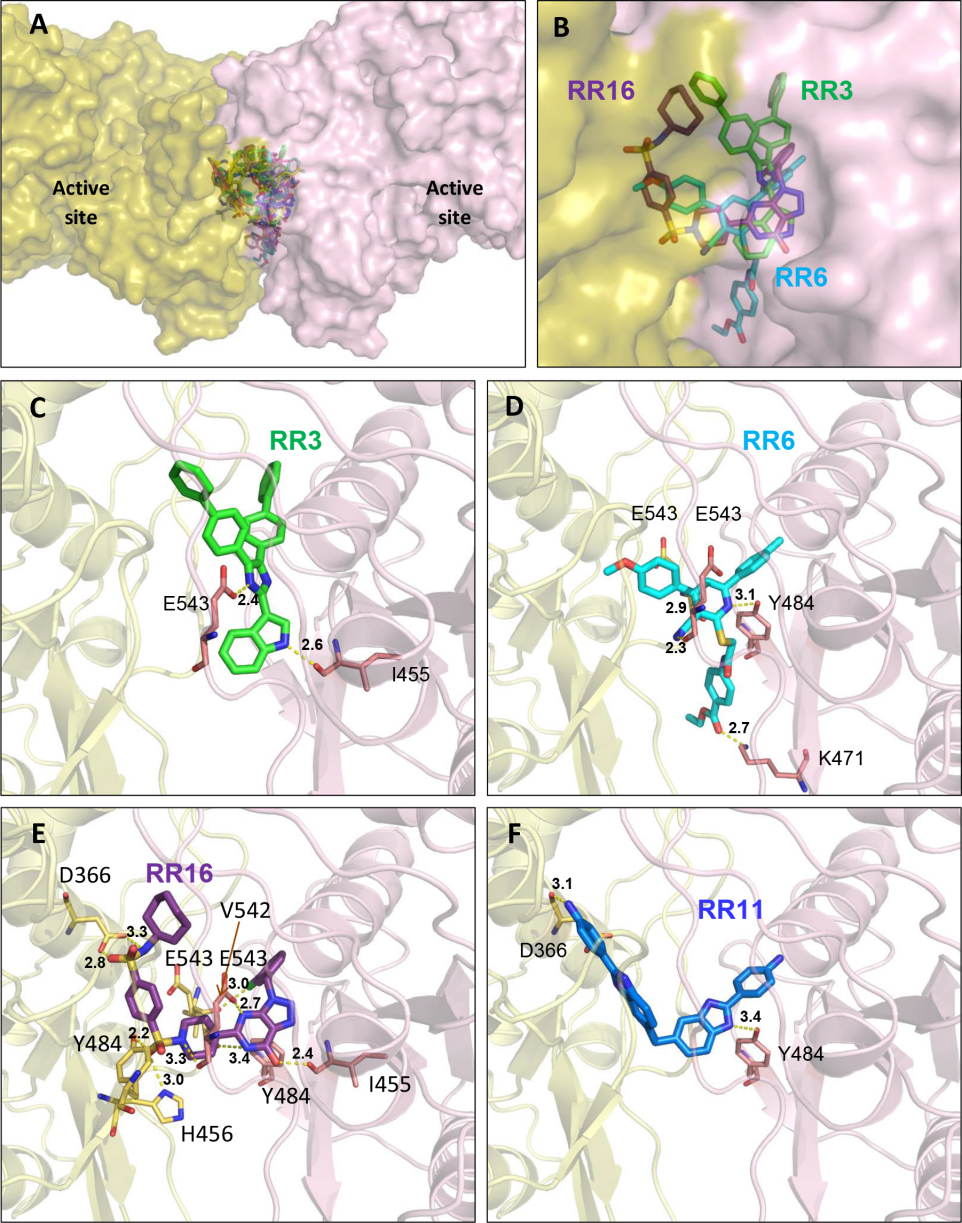
Detailed analysis of the binding mode for best-ranked hit compounds. (A) Overlay of the docking poses from all selected hits at the dimerization interface. Compounds are depicted in sticks and CD73 as solvent accessible surface (yellow and pink for differentiating the two monomers). (B) Overlay of the three most active compounds, **RR3** (green) **RR6** (cyan) and **RR16** (purple). Main polar interactions involved in the binding of **RR3** (C), **RR6** (D) and **RR16** (E) viewed in the same orientation. (F) Binding pose of hit compound **RR11** (blue) holding an extended and dimeric structure.

**Fig 7:**
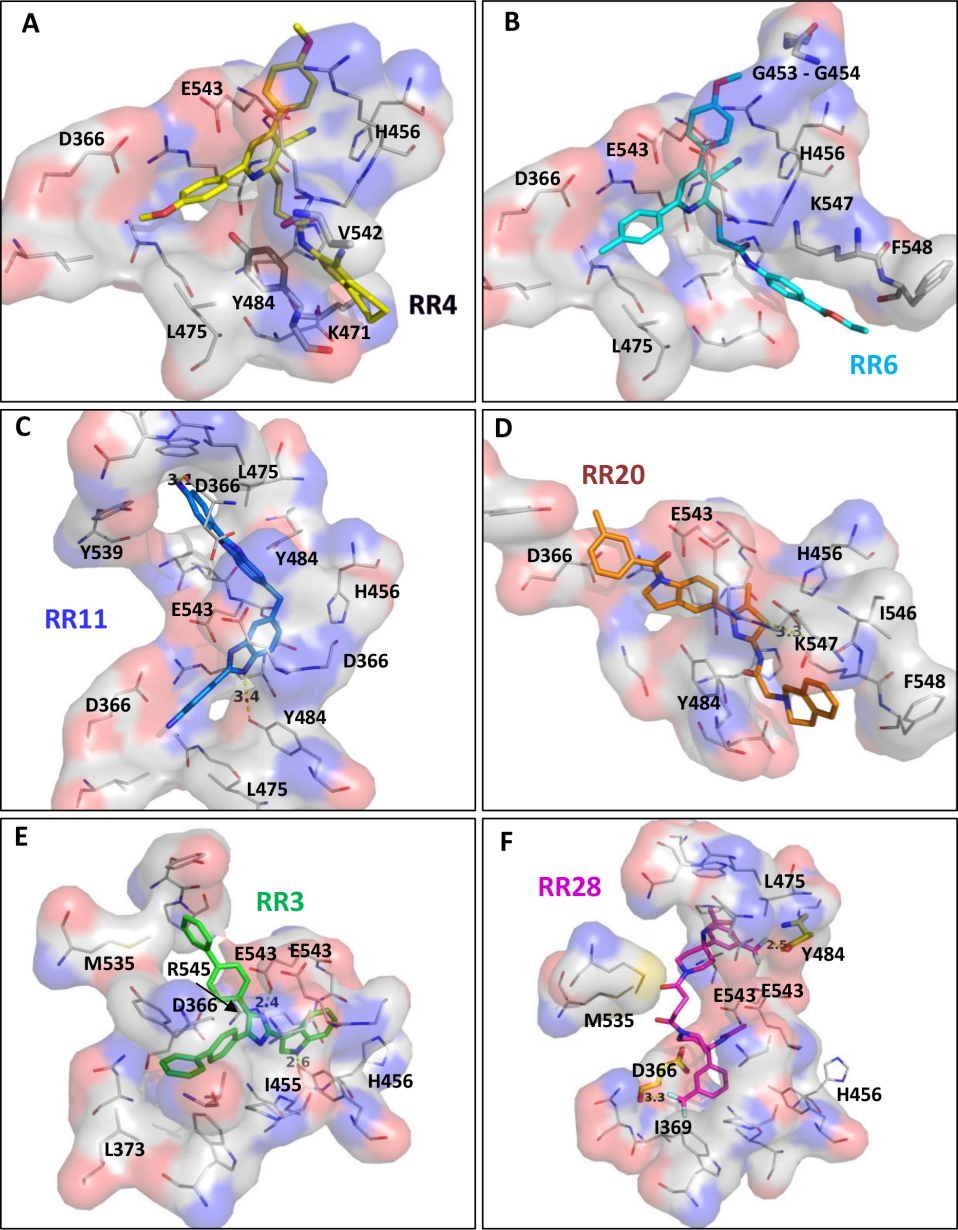
Hydrophobic contacts involved in the binding of RR compounds. Docking poses obtained for the two structurally-related compounds, (A) **RR4** (yellow) and (B) **RR6** (cyan) highlighting the binding differences (thick sticks correspond to residues that are inversely involved). Binding mode of compound having a stretched structure as for **RR11** (C) and for **RR20** (D) depicted as blue and orange sticks, respectively. Comparison of the binding mode for the inhibitory compound **RR3** (E) and the activator **RR28** (F) assuming a common binding site for both. Residues making halogen bonds are depicted in yellow sticks. All residues contributing to hydrophobic contacts (either with backbone or sidechain atoms) are depicted in solvent accessible surface and in thin sticks (all compounds are not oriented identically).

As shown in Fig 6A, selected hit compounds were predicted to bind entirely to the large targeted cavity and they were spanning at least three sub-parts of the cavity (Fig 6B). Focusing on the most active compounds (**RR3**, **RR6** and **RR16**) all of them were deeply buried in the dimer interface and all three hits interact with one or two glutamate residues (E543). However, their binding modes were found to be slightly different. Indeed, for each compound the main interactions with amino acid residues were different, I455 and E543 with **RR3** (Fig 6C), K471 Y484 and E543 (backbone oxygen from both residues from the two monomers) with **RR6** (Fig 6D), D366, I455, H456, Y484 (both) and E543 (both) with **RR16** (Fig 6E). In addition, a halogen bond is formed between V542 backbone oxygen and the chlorine atom of **RR16**. Interestingly, **RR16** was connected to a huge number of amino acid residues in contrast to the other hits. The presence of two sulfone groups may explain this distinct binding. On the other hand, several hit compounds contained a stretched or more spanned chemical structure like **RR11** or **RR20** and were determined as weaker inhibitor than **RR3**. Consequently, a rigid structure may be unfavorable for a tight binding. In addition to its rigidity, **RR11** exhibits a dimeric structure and binds to CD73 with a different orientation compared to **RR3** even though it was found deeply inserted into the dimer interface (Figs 6F and 7C) and making two polar interactions with D366 and Y484.

Since the virtual screening was achieved on five conformers and the docking analysis done on an unique conformer (the main selected one by the ensemble docking and leading to highest computed scores), we analyzed the variations in binding mode for the most interesting compounds depending on the conformer used for docking (S4 Fig). As shown in S4 Fig, slight variations (average RMSD of 0.5 Å) were observed except for **RR3** for which the RMSD was between 1.2 and 1.8 Å. Also, the docking onto conformer C1 often led to a distinct binding in comparison with other conformers. However, the binding mode was found very similar between at least two conformers over the five for the large majority of hit compounds as for **RR4**, **RR6**, **RR9** and **RR16** for instance. The conformers leading to the best scores were C3 and C5 corresponding to the middle and the end of the simulation (from open to closed states). The preferential binding to conformer C5 may be explained by its larger cavity volume and higher hydrophobicity than for C3 suggesting that these properties allowed to afford a stronger binding. It also shows that the binding may be more efficient when the enzyme is closing to form its active site in presence of the substrate. This comparison also indicates that the large collective motion of the enzyme modifies substantially the target cavity in terms of steric space highlighting the importance of using multi-conformational states during the virtual screening.

**Fig 8:**
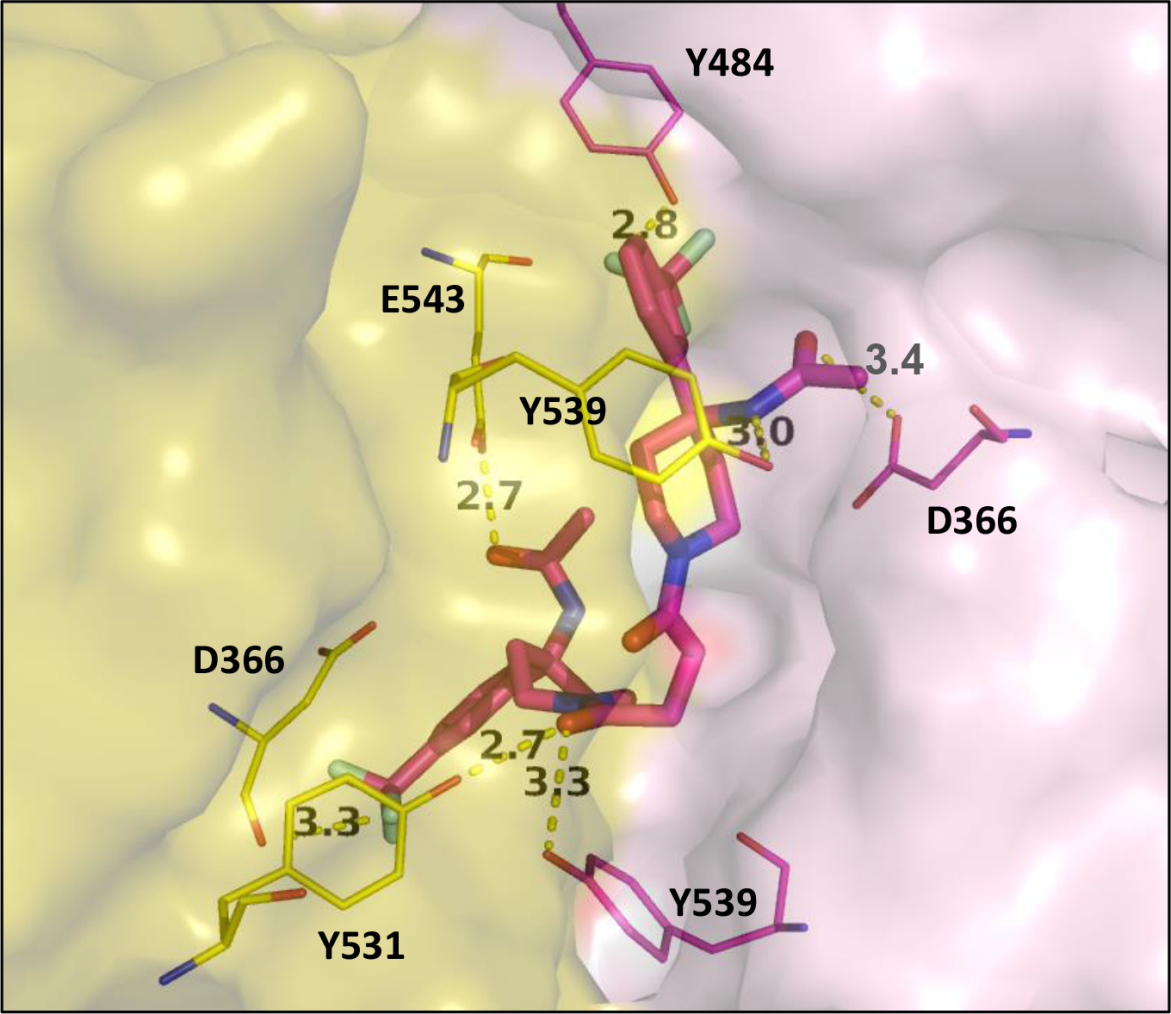
Binding mode of hit compound RR28 linking both enzyme monomers. Residues are depicted in yellow or pink thin sticks according to the monomer they belong and **RR28** in thick pink sticks.

A detailed analysis of the docking binding poses indicates that the hydrophobic contribution in the binding efficiency of hit compounds was quite important as predicted by clog *P* values. Indeed, in addition to the most encountered residues making hydrogen bonds with **RR** compounds (D366, H456, K471, Y484 and E543) numerous apolar residues participated in hydrophobic contacts such as G453-454, I455, L475, V542 and G544. Also, two polar residues, H456 and Y484 are also involved (Figs 7 and 8). Moreover, a few charged residues contributed to these non-bonded interactions such as D366, K471, D473, E543 and R545 reinforcing likely the binding affinity. We first compared the two structurally related compounds **RR4** and **RR6** to highlight their binding mode, orientation and differences (Fig 7A and 7B). These molecules were almost superimposable in their binding site. Highlighting their differences, we observed that the methoxyphenyl group interacts with a patch of glycine residues (G453-G454) for **RR6** and this was not seen with **RR4**. Moreover, the van der Waals contacts involved Y484 and V542 for **RR4** while it was replaced by F548 in the case of **RR6**. This little difference observed in the predicted binding modes may explain the two-fold factor between *K*_i_ values for these two compounds (inhibition constants very close to each other). For elongated and more rigid structures like **RR11** and **RR20** (Fig 7C and 7D), no binding similarities could be observed as one molecule is curved while the other is more stretched allowing to cover a larger surface area in the binding site. This may be explained by their different degree of rigidity. Nevertheless, both compounds connect the two monomers together leading to an enzymatic inhibition. This is achieved through numerous hydrophobic contacts as shown by the interaction with apolar residues. According to their respective *K*_i_ values, a flexible chemical structure seems to be less advantageous for the inhibition efficiency, most probably because of the entropic loss upon binding to CD73.

Interestingly, **RR28** was found to increase the enzyme activity instead of impairing it. To understand the reasons why this molecule to behave as an allosteric activator, we compared its binding to the most potent non-competitive inhibitor, **RR3**. As shown in Fig 7E and 7F, **RR28** did not bind to CD73 in the same orientation and less hydrophobic contacts were found. Two residues (D366 and Y484) are making halogen bonds by interacting with the fluorine atoms. However, apolar residues (I369 and L465) are very close to the fluorine atoms leading to unfavorable contacts. This may explain why the docking score obtained for **RR28** was much lower than that of **RR3** (71 versus 94) and suggests a weak binding in this pocket. Moreover, the structure of this compound shows an axial symmetry enabling to link both monomers of the enzyme through halogen and hydrogen bonds (Fig 8). Therefore, one can imagine that the dimer is better stabilized in the presence of **RR28**. This may explain why **RR28** was found to act as an enhancer of CD73 activity but another explanation would be that it binds to another site to promote such unexpected effect. Nevertheless, two identical residues (D366 and Y539) from each monomer are connecting the hit compound suggesting that this symmetrical compound takes benefit of the symmetry of the dimer.

## Discussion

From this virtual screening study targeting the dimerization interface of CD73 as potential allosteric binding site, several hit compounds were determined as strong non-competitive inhibitors and other as mixed inhibitors. The most active compounds exhibited *K*_i_ values in the low micromolar range allowing for further hit to lead optimization. Structure of hit compounds were characterized by two different scaffolds either as a tripartite shape or an extended structure with similarities within the two families. The large structure allowed spanning the large cavity. Interestingly, some compounds were shown to create a strong linkage between the two monomers leading either to an inhibition or an enhancement of the enzymatic activity. Allosteric regulation has been extensively described for many enzymes, especially kinases like p21-activiated kinase 4 [36], small GTPases [37] or G-protein-coupled receptors for more than half a century [38,39]. All these proteins or enzymes play an important role in maintaining the cell integrity or signaling and have also been pointed out for therapeutic approaches including the development of new cancer treatments. Nowadays, the design of allosteric compounds represent a valuable alternative approach to identify new drugs targeting proteins that are considered “undruggable” by developing either positive or negative allosteric modulators [40]. Within the large Halo-Acid Dehalogenase family from which CD73 belongs, the cytosolic 5’-nucleotidase II is a good example of allosteric regulation by ATP or bisphosphoglycerate as previously described [41,42]. Here, we targeted an interface that is not described as an allosteric site. However, an allosteric activation was observed with **RR28** in addition to the strong inhibition induced by several hit compounds, indicating that the target binding site was able to modulate the enzymatic activity through the binding of small molecules. It must be highlighted here, that the data obtained so far do not allow us to conclude that inhibitors bind actually in the assumed allosteric binding site (targeted during the virtual screening) and this conclusion will only become definitive by solving the crystal structure of the complex, for instance. Similarly, it cannot be excluded that **RR28** binds to a different allosteric pocket to that of **RR3** or **RR4**. Also, another question remains concerning the allosteric effect that includes by definition, a protein conformational change. Here, we could not measure experimentally this effect and we assume that non-competitive inhibitors act as allosteric inhibitors (and the opposite for **RR28** acting as allosteric activator). The chemical nature of the identified compounds leads to high lipophilicity according to their c*log* P values and consequently a lower water solubility. This arises from the selection of hydrophobic compounds present in the chemical library during the screening phase when targeting the interfacial binding site. Screening in the substrate binding site would have selected more hydrophilic compounds. Nevertheless, the current hit compounds will have to be optimized to increase their bioavailability. This step can be achieved by several methods often used in all drug discovery programs (search by similarity or pharmacophore models) and keeping in mind that a certain degree of lipophilicity is required to ensure a tight binding in the dimer interface. Alternatively, permeation enhancers may be useful to improve their physicochemical properties before reaching the enzyme target such as cyclodextrin-based formulations [43–45] or by using chitosan [46] or glyconucleolipid [47,48] derivatives leading to both an increase in bioavailability and half-life of the compound.

The main objective of this study was to block the enzymatic activity by hindering the dynamics of the enzyme that is required for its function (and therefore the active site formation leading to the hydrolysis of AMP into adenosine). One interesting feature here is the selected cavity that is located at the dimer interface and can be therefore considered as a protein-protein interface. This point was already discussed in previous publications targeting protein-protein or protein-DNA interfaces and led to the discovery of interfacial inhibitors, like Brefeldin A binding to the Arf-Sec7 interface or camptothecin binding to topoisomerase I-DNA complex [49].

CD73 has been extensively studied for its implication in cancer development and progression [4] and in addition to the monoclonal antibody (MEDI9447) [29], several small molecule inhibitors have been developed. All these compounds (anthraquinone [50], sulfonic acid or sulfonamide derivatives [51,52] or those being derived from APCP [26]) were designed by targeting the substrate binding site or by analogy to the substrate itself, and most of them act as competitive inhibitors. One exception has been recently described with 2-alkoxy-3-(sulfonylarylaminomethylene)-chroman-4-one derivatives acting as uncompetitive inhibitors [53]. This study indicates that these inhibitors block the enzyme by targeting an enzyme-substrate intermediate of the reaction. This was the first suggestion of the presence of a binding site different to that of the substrate. Here, we describe for the first time the inhibition of CD73 activity by a most likely allosteric mechanism, which may lead to higher enzyme selectivity and less off-target effects.

## Materials and methods

### Molecular dynamics simulations and virtual screening

The overall strategy is schematically illustrated in Fig 1A. Targeted molecular dynamics (TMD) simulations were carried out using two crystal structures of CD73 in the direction from the open (4H2G) to the closed (4H2I) conformation. All calculations were performed with NAMD 2.11 [54] in the isobaric–isothermal ensemble. The pressure (1 atm) and temperature (310 K) were kept constant using Langevin dynamics and Nosé-Hoover Langevin piston [55,56]. All protein atoms and Zn ions were described by the CHARMM27 force field [57]. The substrate AMP was modelled using adenosine structure from 4H2G and inorganic phosphate from 4H1S by structural alignments of respective C-domains. Missing parameters in Charmm force field were added by homology to ADP but with atomic partial charges computed with Gaussian (RHF/6-31G) by fitting the electrostatic potential surface. The system was solvated with explicit water (TIP3P model), neutralized with four sodium ions and replicated in each direction using periodic boundary conditions. The short-range Lennard-Jones potential was smoothly truncated from 10 to 12 Å and the PME (Particle Mesh Ewald) algorithm [58] was used to calculate long-range electrostatics with a grid spacing of 1 Å. The potential energy of the molecular systems was minimized for 100,000 steps of conjugate gradient (time step of 2 fs). After a gradual heating from 0 to 310 K, the two systems were further equilibrated for 100,000 steps. A spring force constant of 200 kcal/mol/A^2^ was applied to all atoms and defined in the TMD potential term (U_TMD_, see Eq 1) allowing reducing the root mean square (RMS) distance between open (4H2G) and closed (4H2I) conformations during 20 ns. The two C-domains (residues 337–549) of both structures were aligned prior to simulation.

$$U_{TMD}=\frac{1}{2}\frac{k}{N}{\left[RMS\left(t\right)-RMS^{*}\left(t\right)\right]}^{2}$$ (Eq 1)

where RMS(t) is the instantaneous best-fit RMS distance of the current coordinates from the target coordinates, and RMS*(t) evolves linearly from the initial RMSD at the first TMD step to the final RMSD at the last TMD step. The elastic constant k is scaled down by the number N of targeted atoms.

Identification and characterization of druggable cavities were achieved with Fpocket or MDpocket software [59]. Selection criteria were the volume and mean local hydrophobic density (ratio of neighboring apolar alpha spheres divided by the total number of apolar alpha spheres in the pocket); this ratio is then normalized in respect to the other binding pockets [60]. The potential energy function of the five conformers selected from TMD simulation (and further used for ensemble virtual screening) was minimized with 50,000 steps of conjugate gradient using NAMD. The conformers were subjected to a careful structural quality assessment using the ProSA-web server [61] (https://prosa.services.came.sbg.ac.at/prosa.php) (S2 Fig) and by computing their Ramachandran diagrams (S3 Fig) by using the Rampage program hosted at the University of Cambridge [62] in order to compare the overall quality with the experimental crystal structure (4H2G and 4H1S). A chemical library of 324,400 compounds was generated from the Molport screening compound database gathering 34 suppliers and composed of natural and synthetic molecules with drug-like properties (http://www.molport.com). The library of screening compounds was composed of unique molecules, commercially available from several main suppliers (Asinex, ChemDiv Inc., Vitas-M laboratory and Enamine). Before using it for virtual screening, the library was filtered in order to remove duplicates, add explicit hydrogens, generate 3D coordinates and finally to transform in PDBQT (Vina) or Sybyl mol2 (Gold) format using Open Babel 2.4.1 [63]. Despite a careful filtering, few compounds escaped to the modified Lipinski’s rules of 5 (initial Ro5 with a molecular weight allowed to be greater than 500 Da and a clog *P* greater than 5) as shown by some low molecular weight fragments found in the library. Virtual screening was performed using the highly parallelized implementation, VinaLC-1.1.2 [64] of the Autodock Vina molecular docking program [65]. A confirmation of the docking poses was achieved using a second program and scoring function (GOLD 5.2 program, CCDC Software Limited, [66]) in order to increase the prediction accuracy. The center of mass of the two E543 residues (from both monomers), located in the vicinity of the interface was targeted with radius of 15 Å around this point. The goldscore function was used to rank the docking solutions by using the clustering method (complete linkage) from the RMSD matrix of solutions. As the conformer C3 (S1 Fig to S3 Fig) was selected most of the time during the ensemble docking with the 5 TMD conformers and led to the highest scores when tested separately, this conformer was kept for the docking analysis of all hit compounds (SAR relationships). Molecular dynamics simulations were analyzed with the VMD software [67] and structural analysis and visualization of docking poses were prepared using the PyMOL Molecular Graphics System (version 1.8, Schrödinger, LLC). For molecular interactions between CD73 and RR compounds, a maximum cutoff distance of 3.5 Å and 4.5 Å was used for hydrogen bonds and van der Waals contacts calculations, respectively. The clog *P* values for RR compounds were calculated using the robust Molinspiration chemoinformatics utility and the mi-log *P* model (www.molinspiration.com). Various ligand efficiency metrics have been computed such as LE for ligand efficiency (LE = [1.4 x (-log *K*_i_)]/N_HA_, where N_HA_ is the number of heavy atoms excluding hydrogens and expressed in kcal.mol^-1^.HA^-1^) [34,68], LLE or ligand-lipophilicity efficiency (LLE = p*K*_i_−cLog *P*, where p*K*_i_ = -log(*K*_i_)), BEI or binding efficiency index (BEI = p*K*_i_ / molecular weight in kDa), SEI or surface-binding efficiency index (SEI = (p*K*_i_) / (Polar surface area /100 Å) [69].

### Recombinant protein expression and purification

The plasmid with *NT5E gene* encoding for the human soluble sCD73 protein (residues 27–549) was kindly provided by Prof. N. Scrutton [31]. This construct already contained a His-tag at the C-terminus and a signal sequence derived from human extracellular glycoprotein (osteonectin, residues 1–19) followed by Leu-Ala-Ser allowing extracellular expression of sCD73 [70]. The insert was subcloned into pFastBac^TM^ vector 1 (ThermoFisher Scientific) after PCR amplification to include *EcoRI/NotI* restriction sites. Protein was expressed in Sf9 insect cells (Life Technologies) using the pFastBac baculovirus system (ThermoFisher Scientific) according to the manufacturer’s instructions. Insect cells were grown in suspension with stirring at 110 rpm in EX-CELL 420 medium at 28°C (Sigma) up to a density of 4×10^6^cells per mL and then infected with baculovirus encoding CD73. The cellular supernatant was harvested by centrifugation (20 min/31,000 g) 48 h post-infection, filtered (0.22 μm), supplemented with protease inhibitors (leupeptin, benzamidine and PMSF at 100 μg/mL) and concentrated on crossflow cassette (Vivaflow 200 Sartorius). The concentrate was centrifuged (30 min/186,000 g) and purified on HisTrap Excel column connected to a FPLC Äkta purifier system (GE Healthcare Life Sciences). The enzyme purity, size and activity were assessed by SDS-PAGE, Western blot and steady-state kinetics with various AMP concentrations. An extinction coefficient of 56,310 l.mol^-1^.cm^-1^ was used for determining protein concentration at 280 nm.

### Enzyme inhibition and steady state kinetics assays

The 33 hit compounds were purchased from MolPort compound order service (www.molport.com) gathering all compounds from various suppliers. The purity and structural integrity of the purchased chemical compounds have been evaluated by NMR and mass spectroscopy (S5 Fig). Adenosine 5′-monophosphate sodium salt (AMP) was purchased from Sigma-Aldrich and adenosine 5’-(α,β-methylene) diphosphate (APCP) used as positive control was synthesized using a previously published procedure [26]. The CD73 nucleotidase activity was determined by steady-state kinetics measuring the adenosine produced upon AMP hydrolysis by CD73 over time. The reaction was carried out in a thermostatically controlled beaker under magnetic stirring at 37°C in a buffer containing Tris-HCl 50 mM pH 7, NaCl 100 mM, MgCl_2_ 1 mM, CaCl_2_ 1 mM. Reaction was allowed to occur upon addition of the substrate and stopped by addition of 10% of perchloric acid every 5 s. The same procedure was repeated in presence of each inhibitor at 5 μM. Reaction products were quantified by HPLC chromatography (Waters Alliance) using a Partisphere 5-SAX column (AIT France) and 10 mM ammonium phosphate buffer pH 5.5 as mobile phase. For non-water soluble compounds, DMSO was used and the final percentage did not exceed 0.5% in order to preserve the full enzyme activity (enzyme tolerance for DMSO was determined up to 2%). The commercial human CD73 enzyme produced in eukaryotic cells (Interchim) was used to compare the kinetics parameters of both batches and to confirm the inhibition promoted by hit compounds.

### Determination of inhibition mode and K_i_

For the most interesting compounds, the inhibition mode was determined by steady state kinetic assays to obtain apparent catalytic (*k*_cat_), Michaelis (*K*_M_) and inhibition (*K*_i_) constants. Recombinant enzyme (2.5 nM), substrate (AMP, at eight different concentrations: 1, 1.5, 2.5, 5, 10, 25, 50 and 100 μM) were mixed in a thermostated beaker at 37°C in the presence or in the absence of inhibitors and reaction was stopped every 5 s by acid quenching before HPLC analysis (as mentioned above). Quantification of adenosine and AMP was achieved by integrating peaks (Empower software, Waters) and raw data were analyzed using Grafit 7 (Erithacus software) and fitted with four different equations describing either a competitive, uncompetitive, non-competitive or mixed inhibition mode. The best model (with the lowest Chi square value) fitting the experimental data was considered as the inhibition mode and used for determining *K*_i_. All experiments were carried out using three different inhibitor concentrations and Lineweaver-Burk plots were drawn to illustrate the inhibition modes.

## Supporting information

S1 Table Full-list of the hit compounds selected by virtual screening. Compound name with MolPort code and chemical structure of **RR** compounds ranked by docking score. clog *P* value, enzymatic inhibition of CD73 activity by **RR** compounds (5 μM final concentration) on the purified recombinant enzyme (means +/- SD of three independent experiments) and inhibition constants (*K*_i_) and mode for most active compounds (NC for non-competitive) are indicated. In addition to clog *P*, different metrics for ligand efficiency are included: LE, LLE, BEI and SEI (see Materials and Methods section for details). The five last compounds correspond to smaller hit fragments. Most active hits shown in Fig 2 are highlighted in grey. *means +/- SD of three independent experiments.
(DOCX)

S1 Movie Movie retracing the targeted molecular dynamics simulation. Large collective motions observed during the dynamics of CD73 from the open to the closed conformation. The volume changes observed for targeted cavity are shown in mesh representation.
(MPG)

S1 Fig Conformers characterization by comparison to the crystal structures. **A**) Root mean square deviation (RMSD in Å) of backbone atoms from C-domains between conformers issued from targeted molecular dynamics simulations and crystal structures (C-domains were defined by residues 337–549). **B**) Overlay of crystal structures (4H2G—open state, cyan; 4H1S—closed state, orange) and conformers (C1, yellow; C2, red; C3, blue; C4, magenta and C5, grey). All structures were aligned onto both C-domains of 4H2G. **C**) Zoom-in view of the superimposed C-domains from all modeled and x-ray structures (the targeted cavity at the dimerization site is depicted in grey mesh).
(TIF)

S2 Fig Structure quality assessment using ProSA II Z-score calculation. Structure quality assessment using ProSA II Z-score calculation (Z-score profile is computed using X-ray and NMR references structures and black circle indicates CD73 crystal structure or conformers issued from TMD simulation).
(TIF)

S3 Fig Ramachandran diagrams of crystal structures and conformers issued from TMD simulation. Diagrams were computed using the Rampage program [62]. Percentage of residues found in outlier regions was 0.4% (Q88, P160 and T336) for crystal structures and comprised between 0.9 and 1.6% for the five conformers (C1 to C5). Most of the concerned residues are located in the substrate binding site (H118, P141, P222 and H243).
(TIF)

S4 Fig **Comparison of the docking poses obtained with the five conformers for the most active compounds:** (**A**) **RR3**, (**B**) **RR4**, (**C**) **RR6**, (**D**) **RR9**, (**E**) **RR11**, (**F**) **RR16**, (**G**) **RR18** and (**H**) **RR20**. The binding pose of each compound is shown in stick representation with a color code according to the conformer used for docking (blue for C1, green for C2, yellow for C3, pink for C4 and orange for C5).
(PDF)

S5 Fig NMR and mass spectroscopy data of hit compounds purchased from Molport. (TIF)
