# Supplementary material for: Identification of allosteric inhibitors of the ecto-5'-nucleotidase (CD73) targeting the dimer interface
Source: PLoS Comput Biol. 2018 Jan 29;14(1):e1005943. doi: 10.1371/journal.pcbi.1005943 (PMC5805337; doi:10.1371/journal.pcbi.1005943)
Supplement: S1 Table — Compound name with MolPort code and chemical structure of RR compounds ranked by docking score. clog P value, enzymatic inhibition of CD73 activity by RR compounds (5 μM final concentration) on the purified recombinant enzyme (means +/- SD of three independent experiments) and inhibition constants (Ki) and mode for most active compounds (NC for non-competitive) are indicated. In addition to clog P, different metrics for ligand efficiency are included: LE, LLE, BEI and SEI (see Materials and Methods section for details). The five last compounds correspond to smaller hit fragments. Most active hits shown in Fig 2 are highlighted in grey. *means +/- SD of three independent experiments. (DOCX) [file pcbi.1005943.s001.docx]

**Table S1.** Full list of hit compounds identified by virtual screening on the CD73 dimer interface.

| **Compound**  (code) | **Structure** | **Docking score** | **logP** | **Inhibition (%)*** | **LE / LLE** | **BEI / SEI** | **K_i_ (µM)** | **Mode** |
| --- | --- | --- | --- | --- | --- | --- | --- | --- |
| **RR1**  (MolPort-000-030-642) |  | 96.1 | 3.4 | -14 ± 7 | - | - | - | - |
| **RR2**  (MolPort-000-034-539) |  | 96.1 | 6.6 | 64 ± 13 | 0.16; 0.18 / 0.32; 0.78 | 7.74; 8.41  /  3.46; 3.76 | 4.9 ± 1.7  1.7 ± 0.2 | Mixed |
| **RR3**  (MolPort-001-732-635) |  | 94.0 | 8.7 | 93 ± 5 | 0.23 /  -2.48 | 12.87 /  14.11 | 0.53 ± 0.02 | NC |
| **RR4**  (MolPort-000-007-519) |  | 93.5 | 6.9 | 73 ± 4 | 0.21  /  -0.59 | 10.45  /  5.48 | 1.20 ± 0.08 | NC |
| **RR5**  (MolPort-003-934-255) |  | 93.0 | 8.2 | -10 ± 15 | - | - | - | - |
| **RR6**  (MolPort-000-006-845) |  | 91.3 | 6.6 | 80 ± 0.5 | 0.22  /  -0.64 | 11.47  /  6.1 | 0.68 ± 0.05 | NC |
| **RR7**  (MolPort-002-510-980) |  | 91.0 | 6.0 | 56 ± 12 | - | - | - | - |
| **RR8**  (MolPort-002-696-968) |  | 91.0 | 6.6 | 68 ± 14 | 0.16  /  -1.82 | 7.99  /  4.37 | 17.2 ± 2.5 | NC |
| **RR9**  (MolPort-008-326-250) |  | 91.0 | 6.1 | 65 ± 9 | 0.22  /  -0.53 | 11.68  /  6.97 | 2.7 ± 0.3 | NC |
| **RR10**  (MolPort-007-735-996) |  | 90.0 | 4.4 | 17 ± 13 | 0.21  /  0.64 | 10.46  /  5.28 | 9.39 ± 0.62 | NC |
| **RR11**  (MolPort-001-991-763) |  | 90.0 | 5.2 | 78 ± 8 | 0.22  /  0.01 | 12.11  /  4.77 | 6.1 ± 0.6 | NC |
| **RR12**  (MolPort-000-479-501) |  | 88.0 | 3.7 | -36 ± 1 | - | - | - | - |
| **RR13**  (MolPort-000-109-139) |  | 87.7 | 6.3 | 35 ± 2 | - | - | - | - |
| **RR14**  (MolPort-000-043-449) |  | 87.1 | 1.7 | -36 ± 4 | - | - | - | - |
| **RR15**  (MolPort-000-026-551) |  | 86.3 | 2.6 | 17 ± 0.5 | - | - | - | - |
| **RR16**  (MolPort-000-103-196) |  | 86.3 | 6.7 | 63 ± 1 | 0.20; 0.22  /  1.54; 1.97 | 9.6; 10.25  /  3.95; 4.22 | 0.46 ± 0.10  1.7 ± 0.2 | Mixed |
| **RR17**  (MolPort-000-010-877) |  | 84.0 | 6.4 | 27 ± 6 | - | - | - | - |
| **RR18**  (MolPort-000-274-087) |  | 83.2 | 3.1 | 67 ± 2 | 0.22  /  0.04 | 11.53  /  5.38 | 1.3 ± 0.4 | NC  or mixed |
| **RR19**  (MolPort-000-470-563) |  | 80.1 | 4.0 | 37 ± 8 | - | - | - | - |
| **RR20**  (MolPort-000-117-304) |  | 79.2 | 6.3 | 74 + 1 | 0.22  /  0.53 | 11.31  /  9.02 | 1.22 ± 0.11 | NC |
| **RR21**  (MolPort-000-250-727) |  | 79.0 | 4.7 | 61 ± 4 | - | - | Not soluble | Un-determined |
| **RR22**  (MolPort-000-377-970) |  | 78.2 | 5.7 | 22 ± 5 |  |  | - | - |
| **RR23**  (MolPort-000-117-322) |  | 77.0 | 4.9 | 56 ± 3 | - | - | - | - |
| **RR24**  (MolPort-000-471-309) |  | 75.5 | 2.9 | -7 ± 8 |  |  | - | - |
| **RR25**  (MolPort-000-517-582) |  | 75.3 | 3.0 | -16 ± 4 | - | - | - | - |
| **RR26**  (MolPort-000-238-978) |  | 74.2 | 5.1 | 53 ± 3 | - | - | - | - |
| **RR27**  (MolPort-000-103-199) |  | 72.9 | 5.1 | -16 ± 14 | - | - | - | - |
| **RR28**  (MolPort-000-113-705) |  | 71.1 | 5.8 | -61 ± 10 | - | - | - | - |
| **RR29**  (MolPort-028-812-821) |  | 66.0 | 1.6 | 18 ± 3 | - | - | - | - |
| **RR30**  (MolPort-001-598-937) |  | 66.0 | 0.0 | 10 ± 5 | - | - | - | - |
| **RR31**  (MolPort-015-137-484) |  | 64.0 | 1.8 | -22 ± 5 | - | - | - | - |
| **RR32**  (MolPort-001-932-876) |  | 53.0 | 2.9 | 14 ± 8 | - | - | - | - |
| **RR33**  (MolPort-007-851-347) |  | 51.0 | 2.6 | 55 ± 12 | - | - | - | - |
