# Supplementary figures and images for: Identification of allosteric inhibitors of the ecto-5'-nucleotidase (CD73) targeting the dimer interface
Source: PLoS Comput Biol. 2018 Jan 29;14(1):e1005943. doi: 10.1371/journal.pcbi.1005943 (PMC5805337; doi:10.1371/journal.pcbi.1005943)

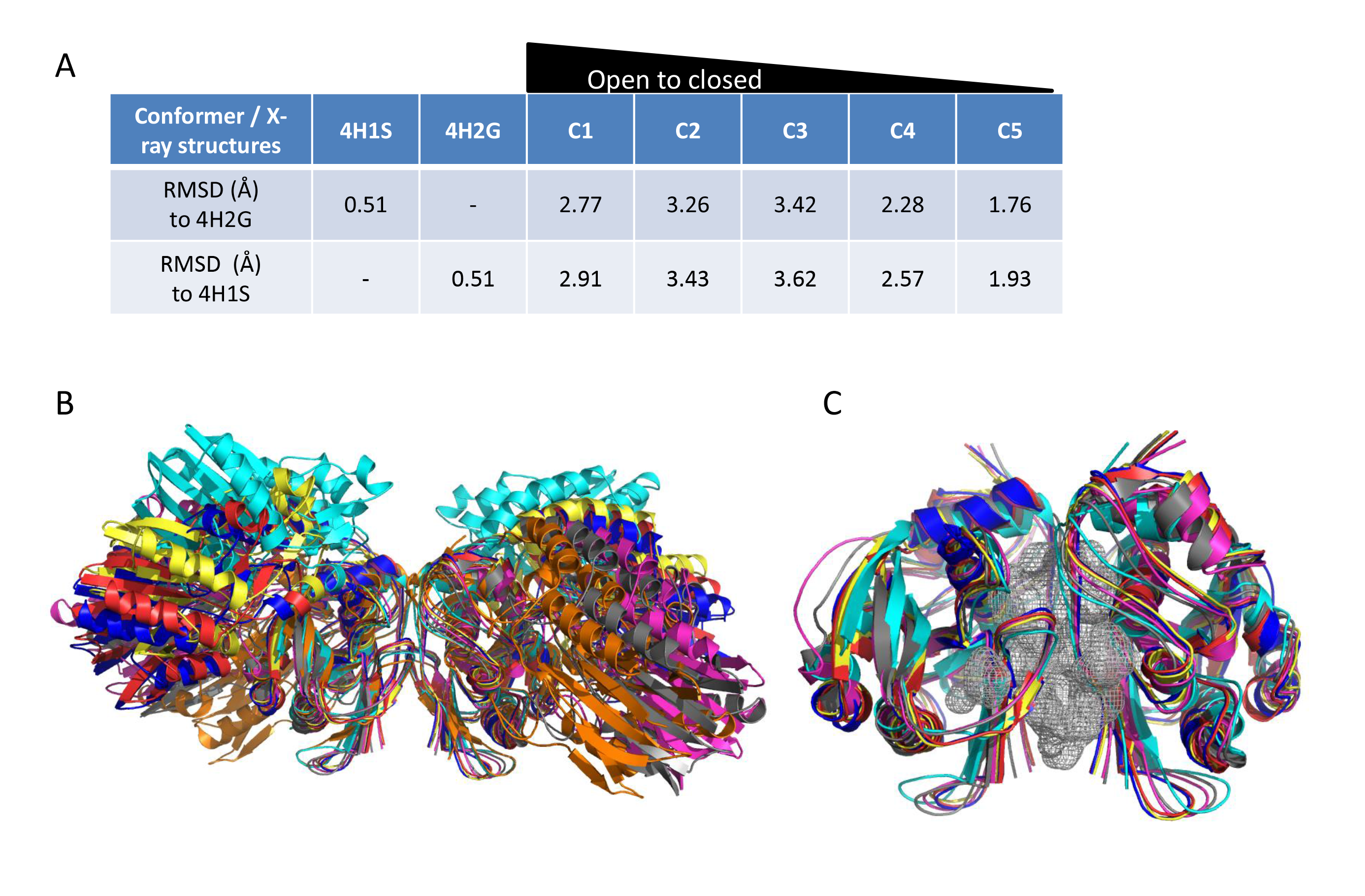

Supplement: S1 Fig — A) Root mean square deviation (RMSD in Å) of backbone atoms from C-domains between conformers issued from targeted molecular dynamics simulations and crystal structures (C-domains were defined by residues 337–549). B) Overlay of crystal structures (4H2G—open state, cyan; 4H1S—closed state, orange) and conformers (C1, yellow; C2, red; C3, blue; C4, magenta and C5, grey). All structures were aligned onto both C-domains of 4H2G. C) Zoom-in view of the superimposed C-domains from all modeled and x-ray structures (the targeted cavity at the dimerization site is depicted in grey mesh). (TIF) [file pcbi.1005943.s003.tif]

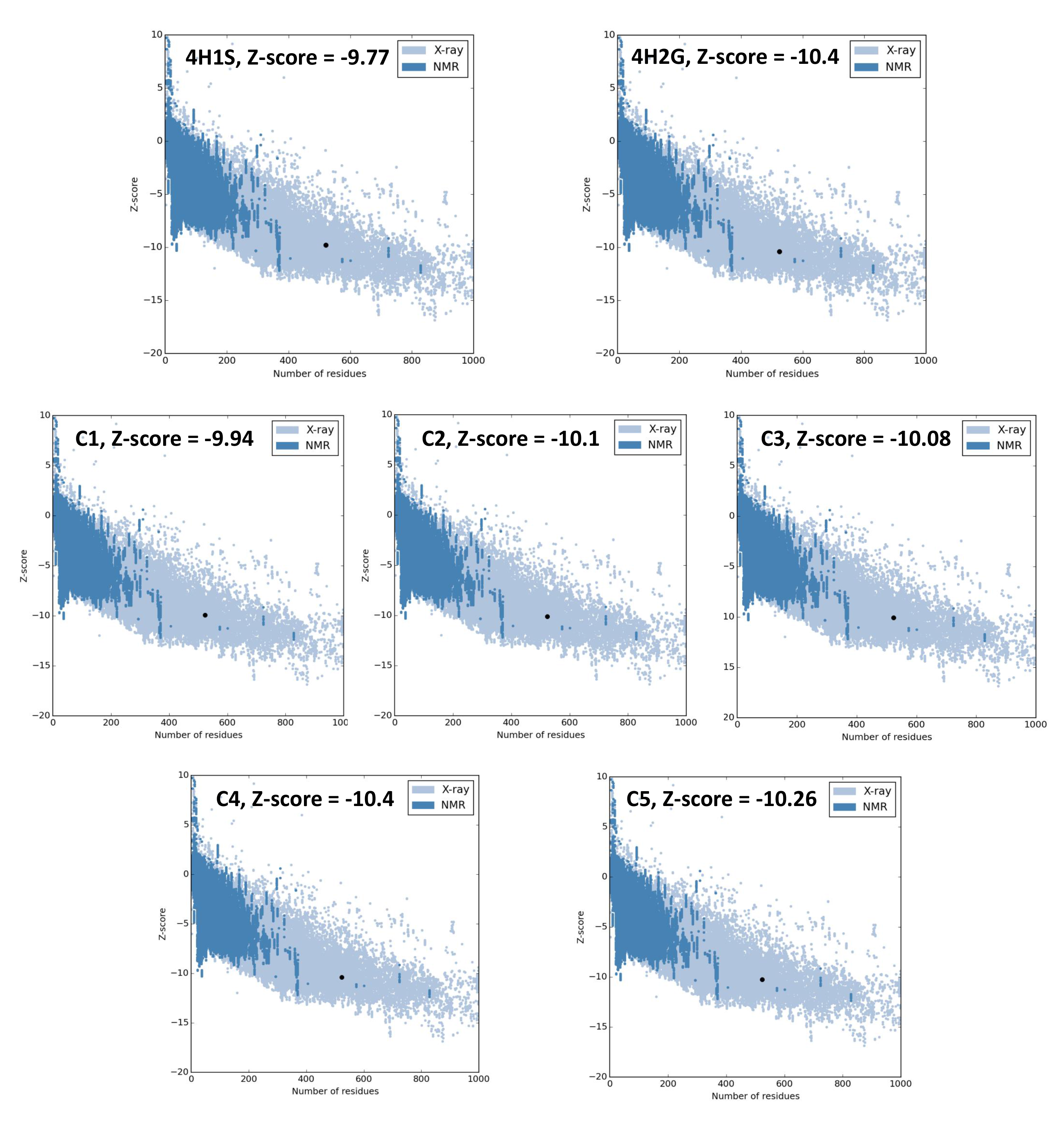

Supplement: S2 Fig — Structure quality assessment using ProSA II Z-score calculation (Z-score profile is computed using X-ray and NMR references structures and black circle indicates CD73 crystal structure or conformers issued from TMD simulation). (TIF) [file pcbi.1005943.s004.tif]

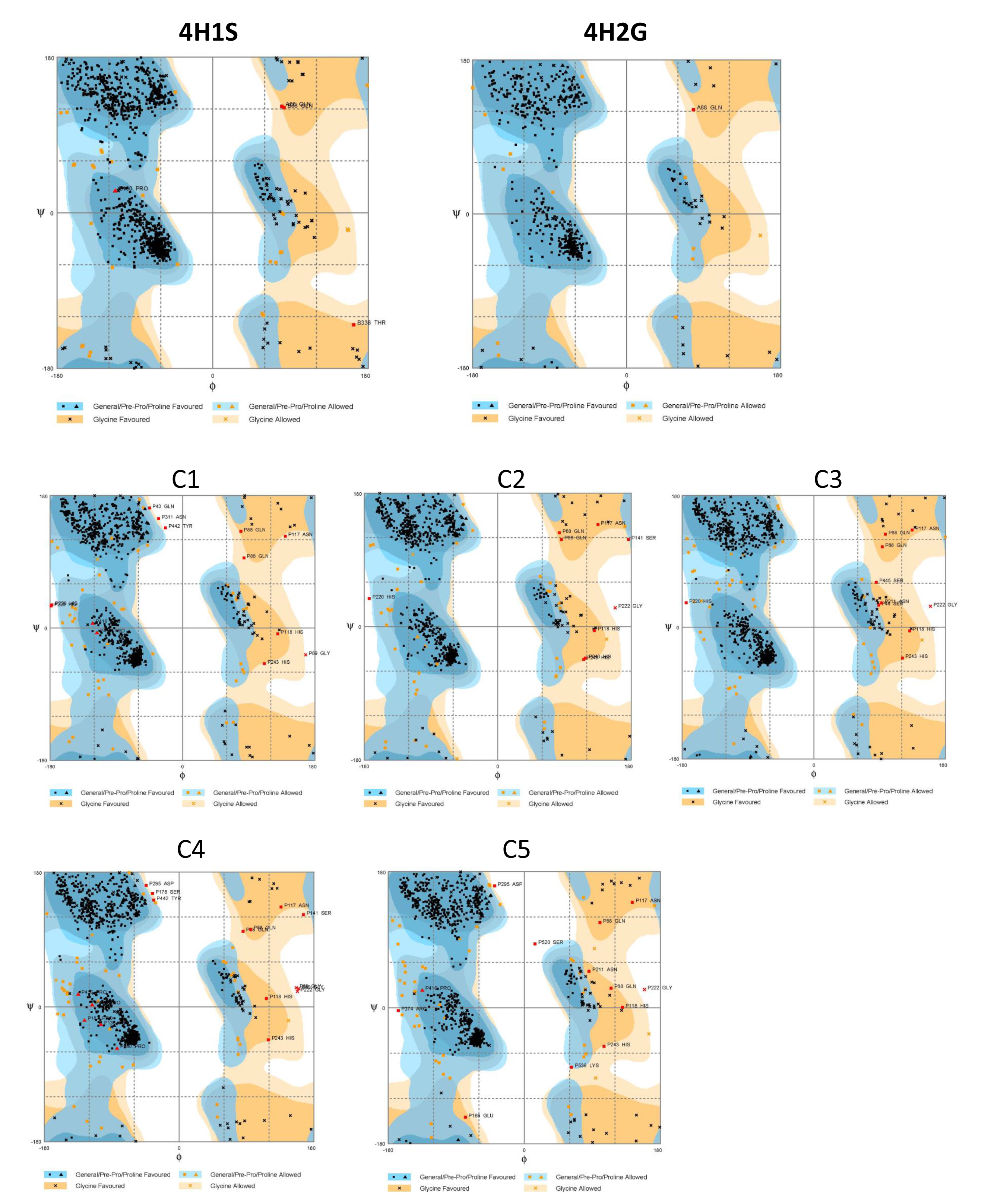

Supplement: S3 Fig — Diagrams were computed using the Rampage program [62]. Percentage of residues found in outlier regions was 0.4% (Q88, P160 and T336) for crystal structures and comprised between 0.9 and 1.6% for the five conformers (C1 to C5). Most of the concerned residues are located in the substrate binding site (H118, P141, P222 and H243). (TIF) [file pcbi.1005943.s005.tif]

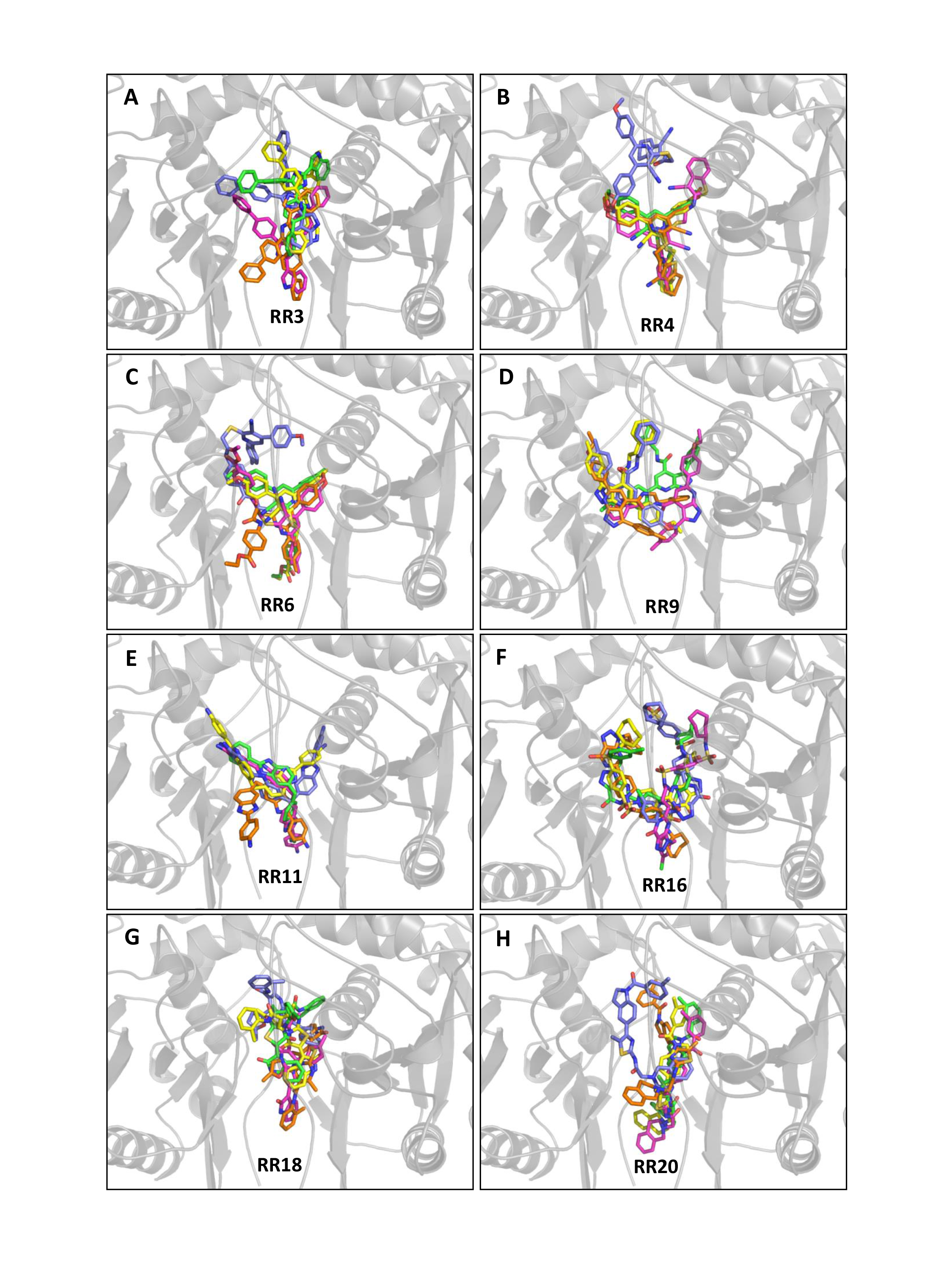

Supplement: S5 Fig — (TIF) [file pcbi.1005943.s007.tif]
