# Supplementary material for: Identification of allosteric inhibitors of the ecto-5'-nucleotidase (CD73) targeting the dimer interface
Source: PLoS Comput Biol. 2018 Jan 29;14(1):e1005943. doi: 10.1371/journal.pcbi.1005943 (PMC5805337; doi:10.1371/journal.pcbi.1005943)

# Copies of $^1$H NMR and MS Spectra of RR compounds

## *RR2 (MolPort-000-034-539)*

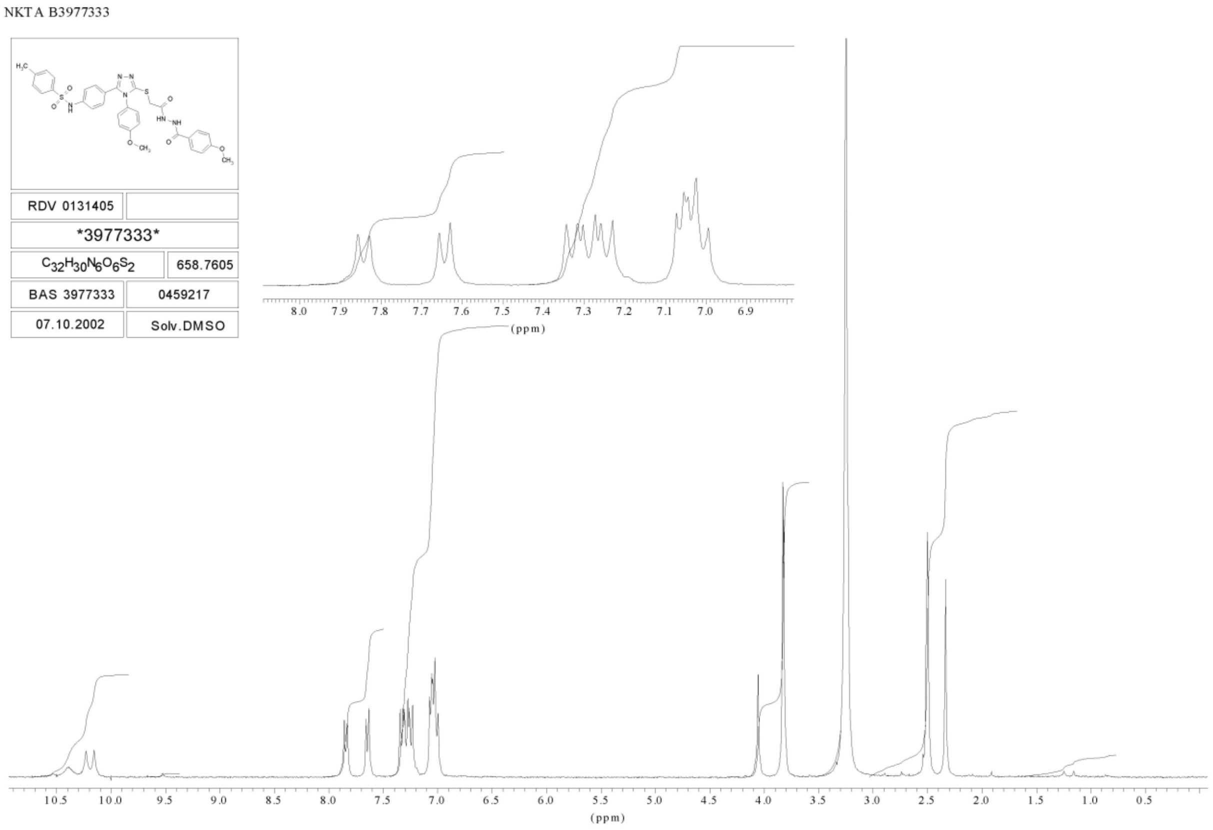


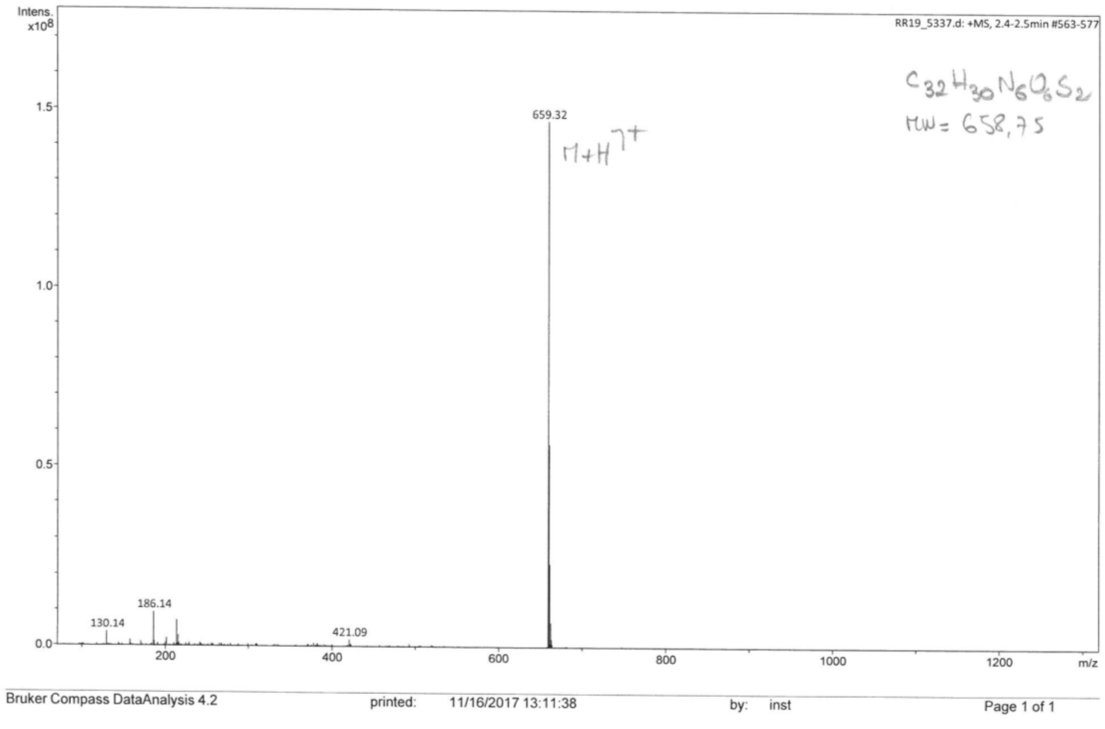

## *RR3 (MolPort-001-732-635)*

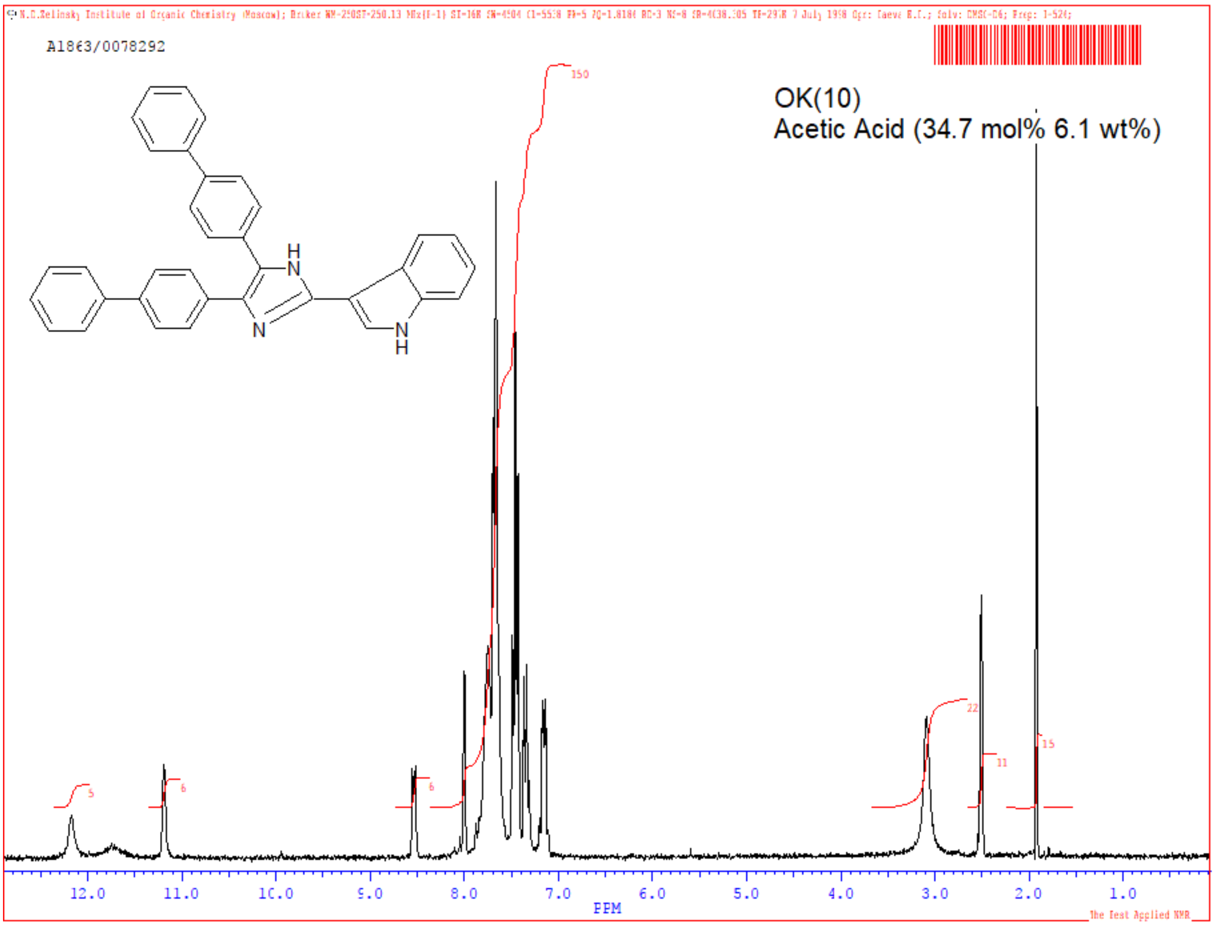


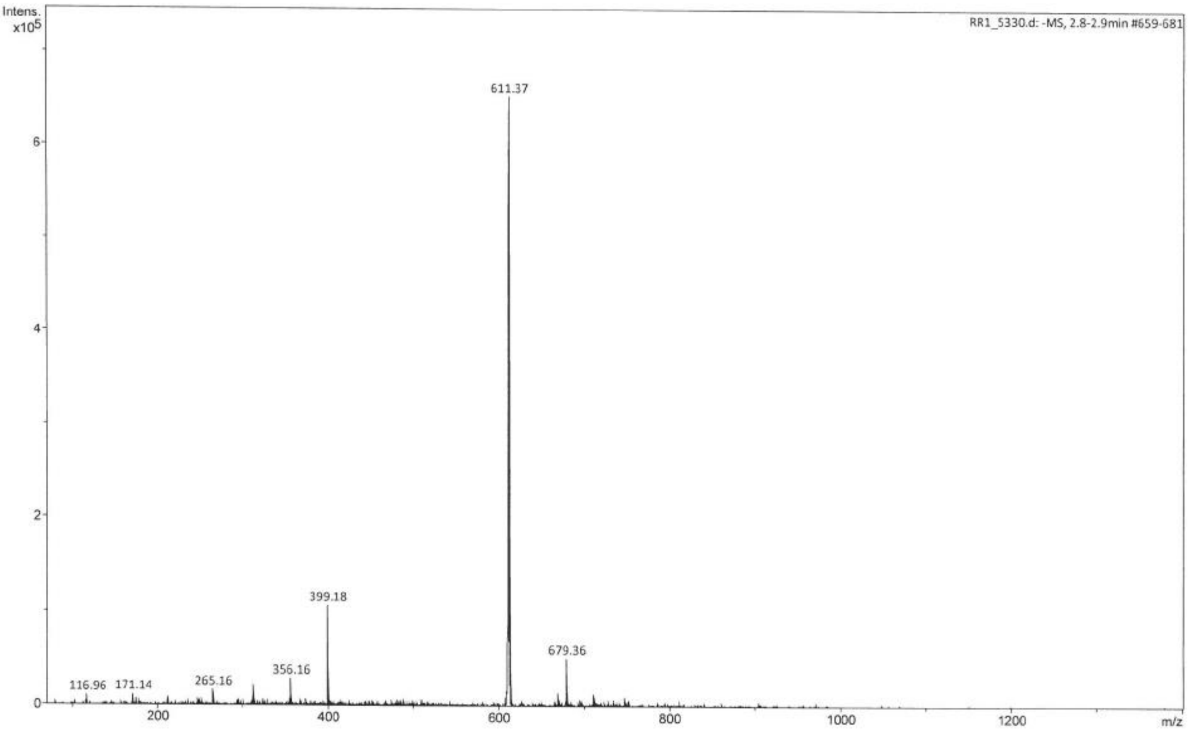

*RR4 (MolPort-000-007-519)*

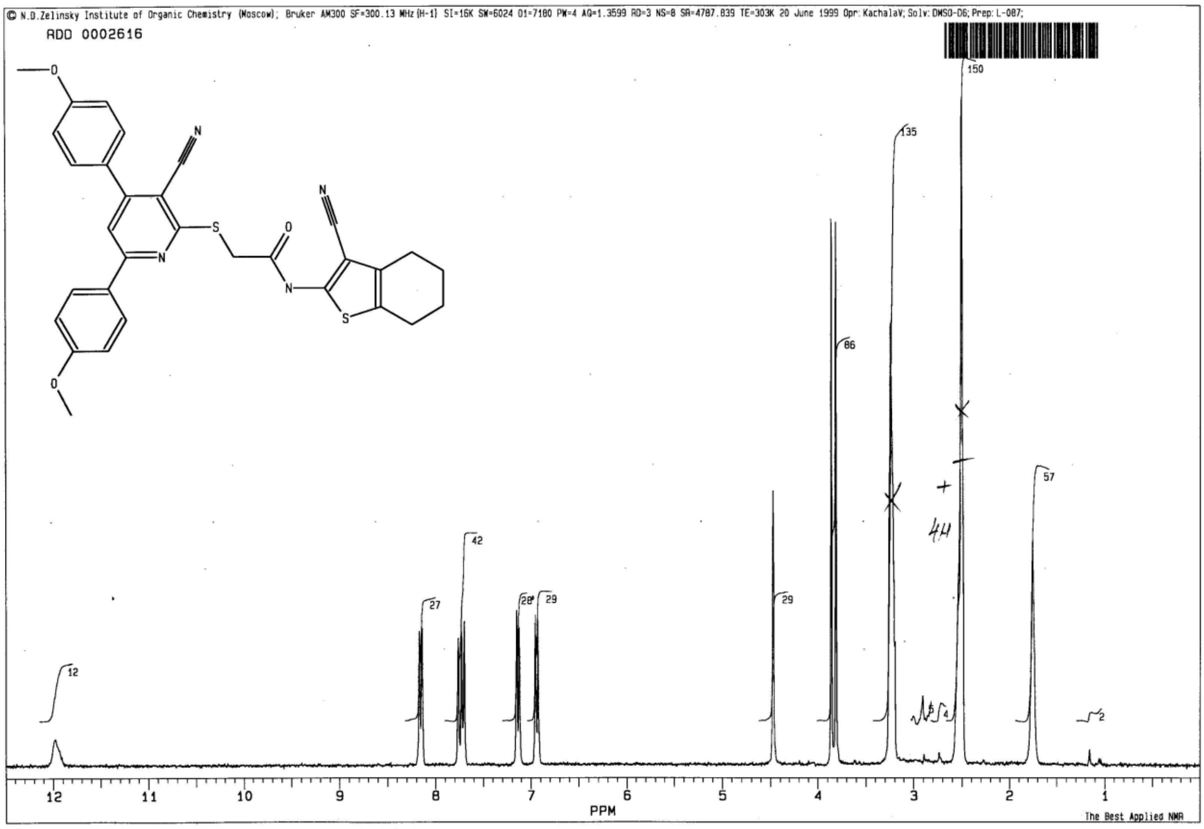


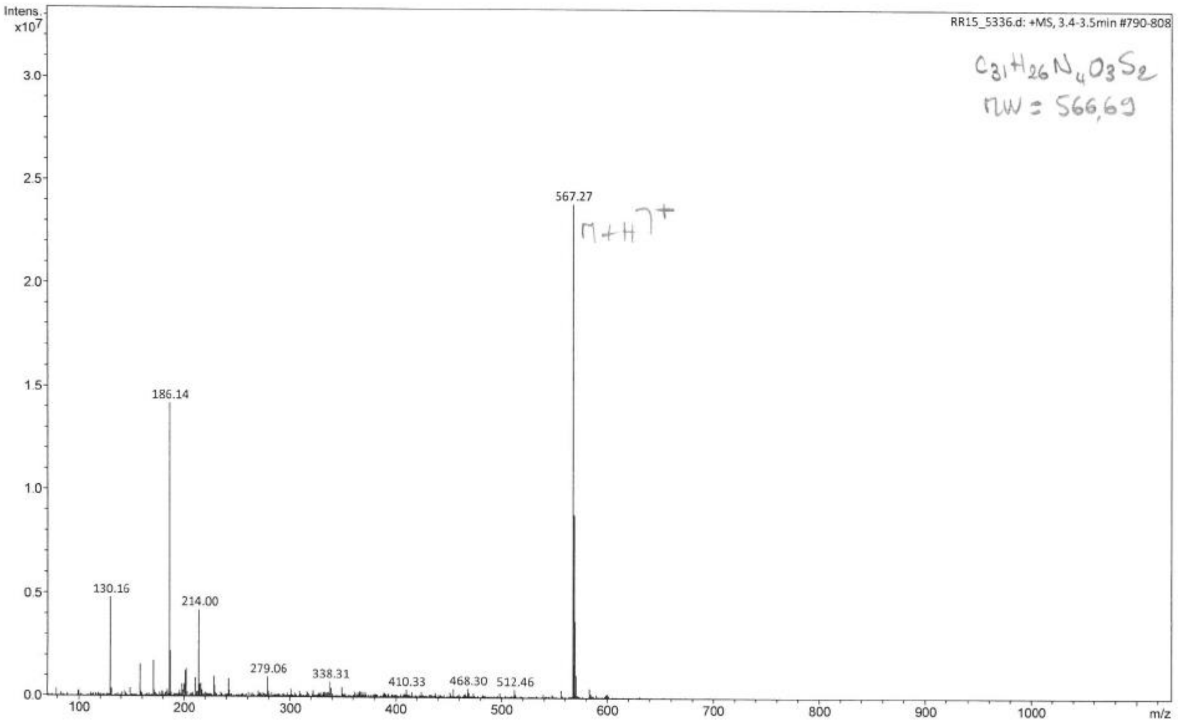

***RR6 (MolPort-000-006-845)***

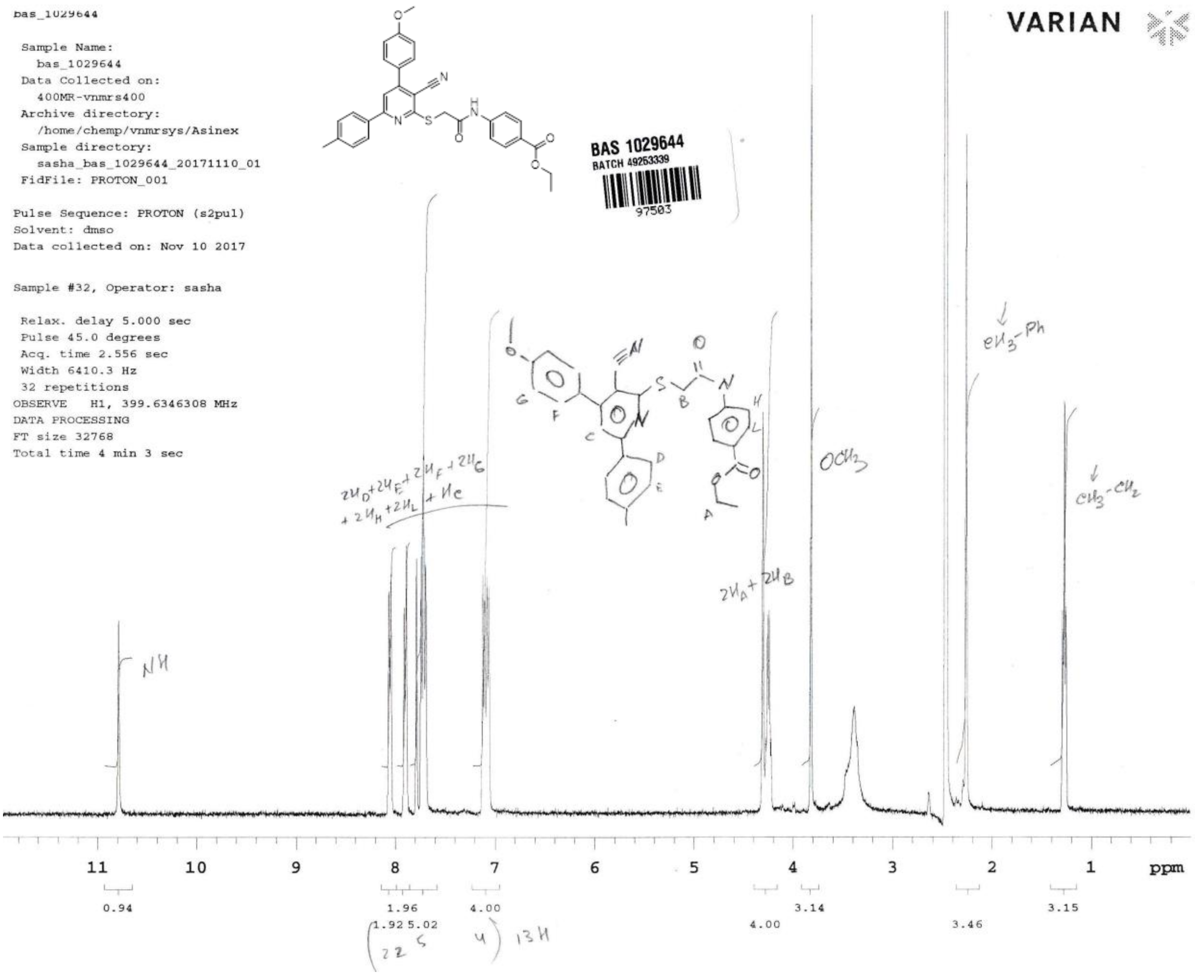


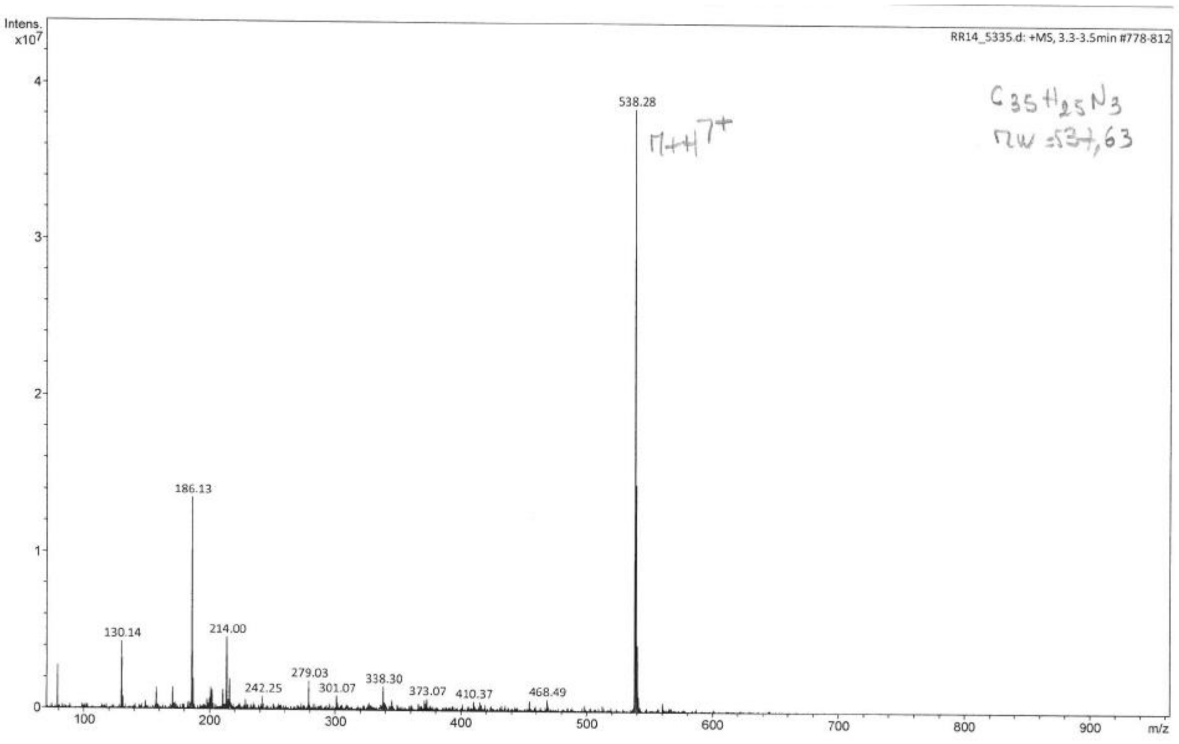

## *RR8 (MolPort-002-696-968)*

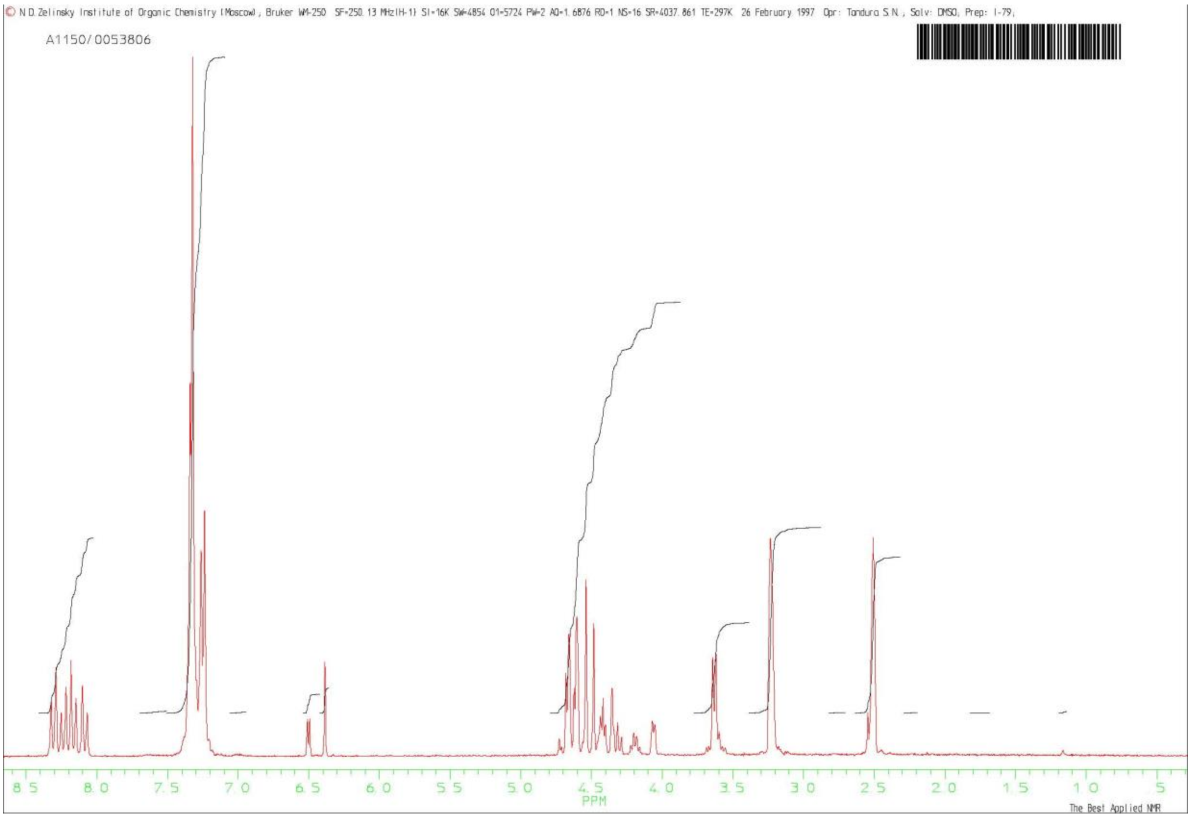


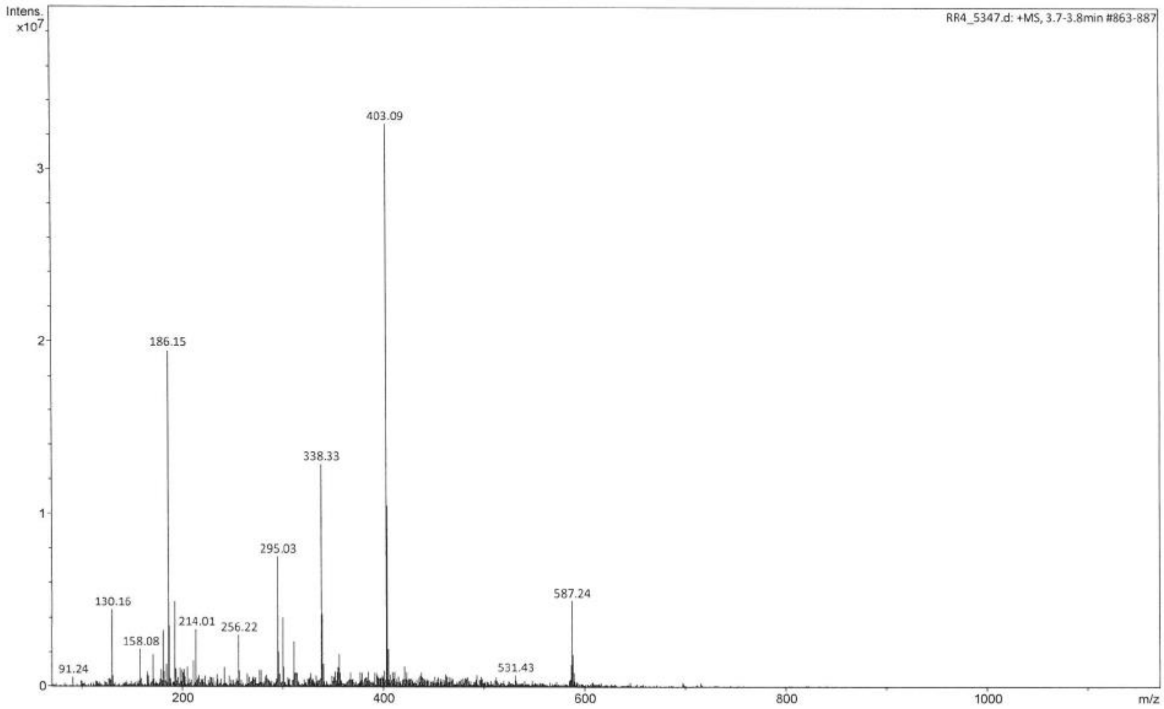

## *RR9 (MolPort-008-326250)*

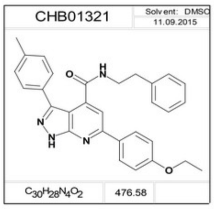


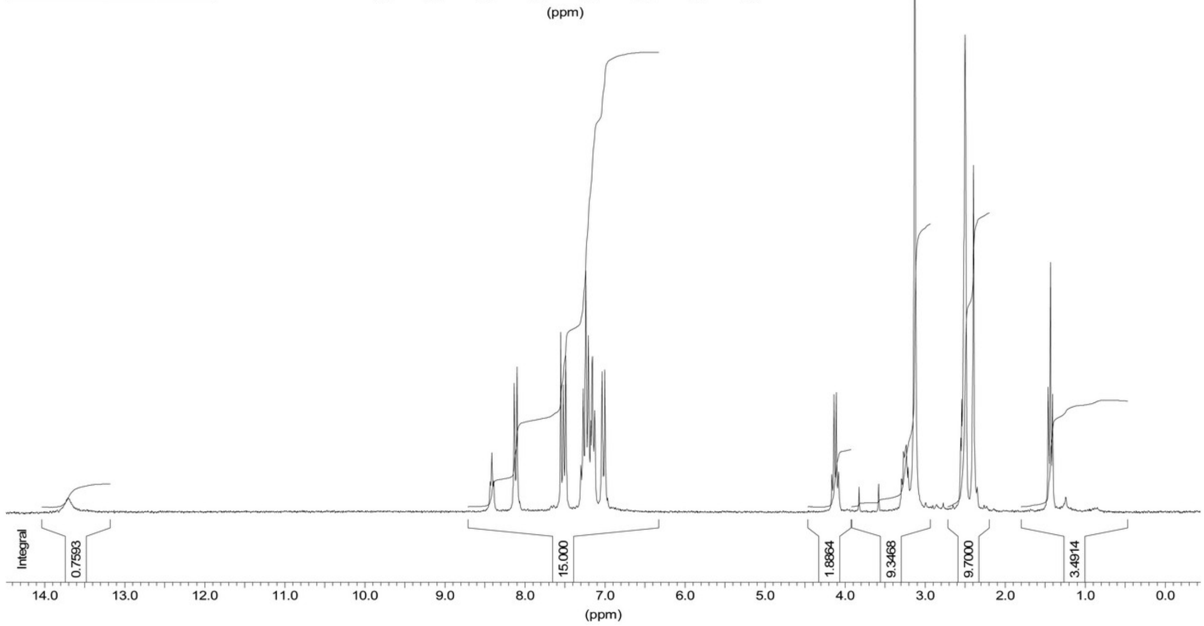


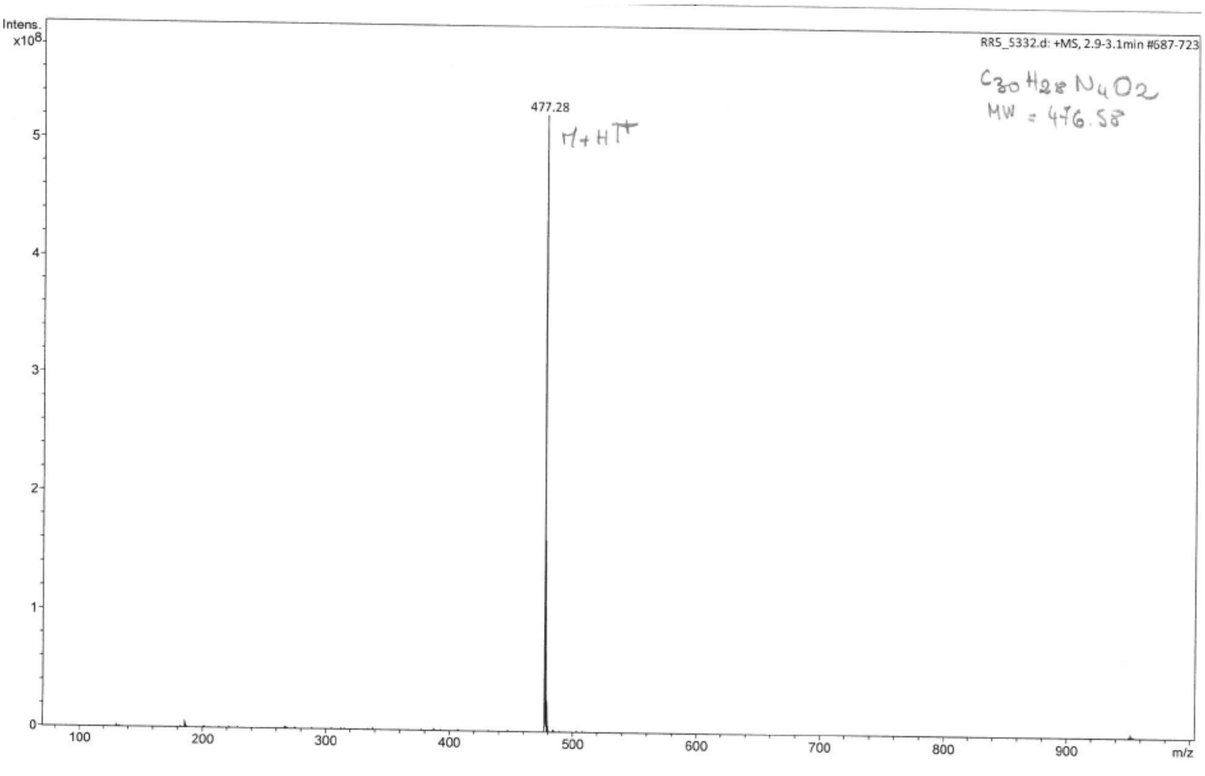

*RR11 (MolPort-001-991-763)*

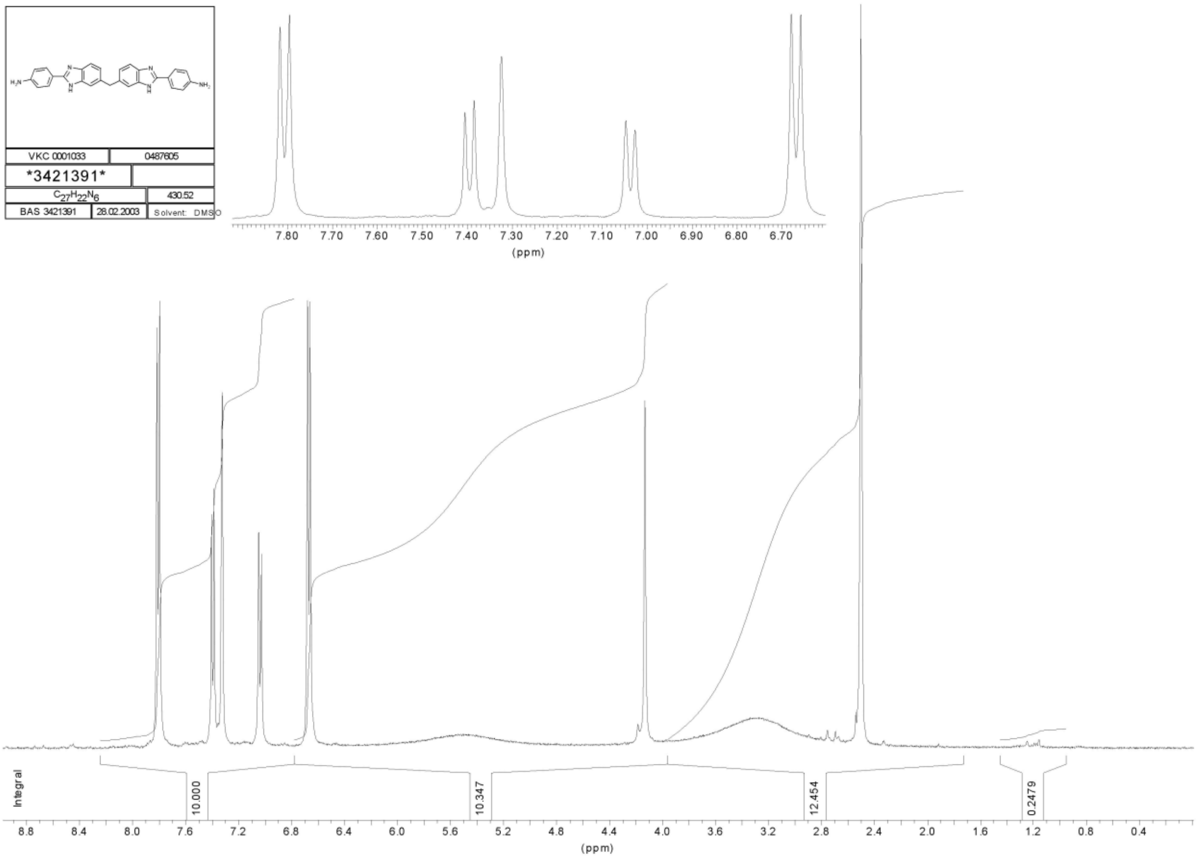


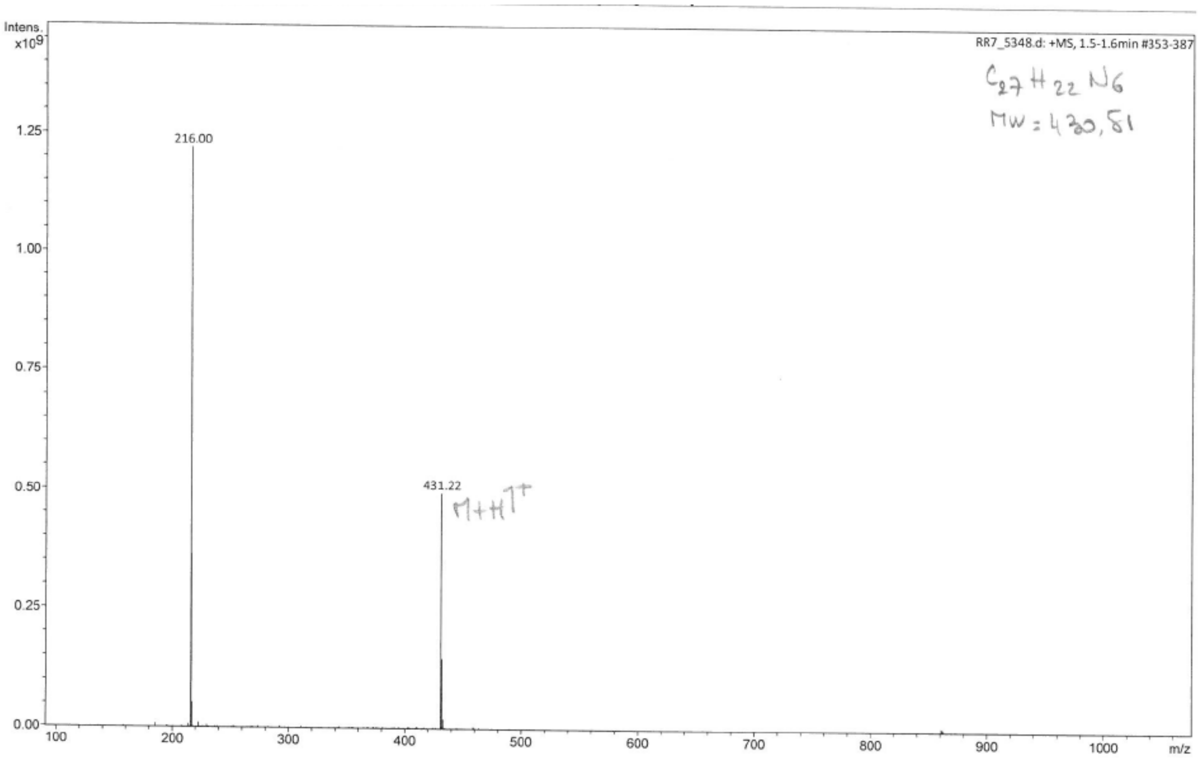

***RR16 (MolPort-000-103-196)***

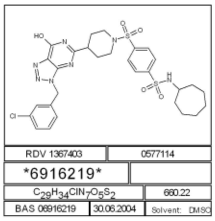


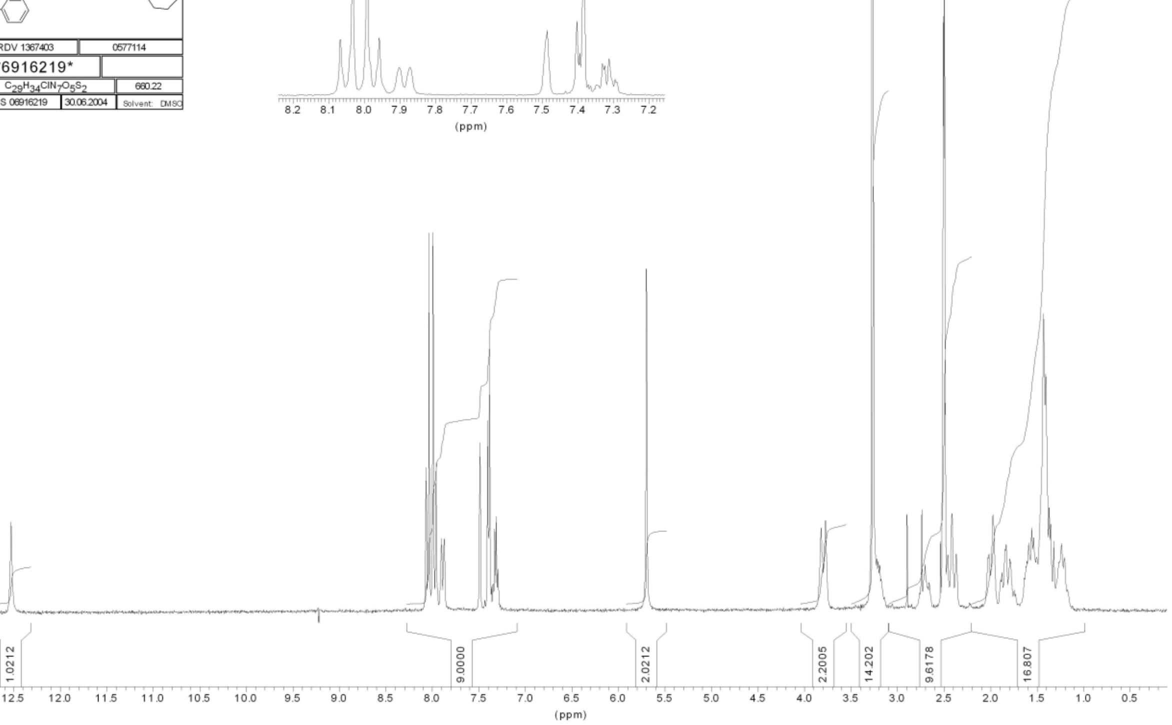


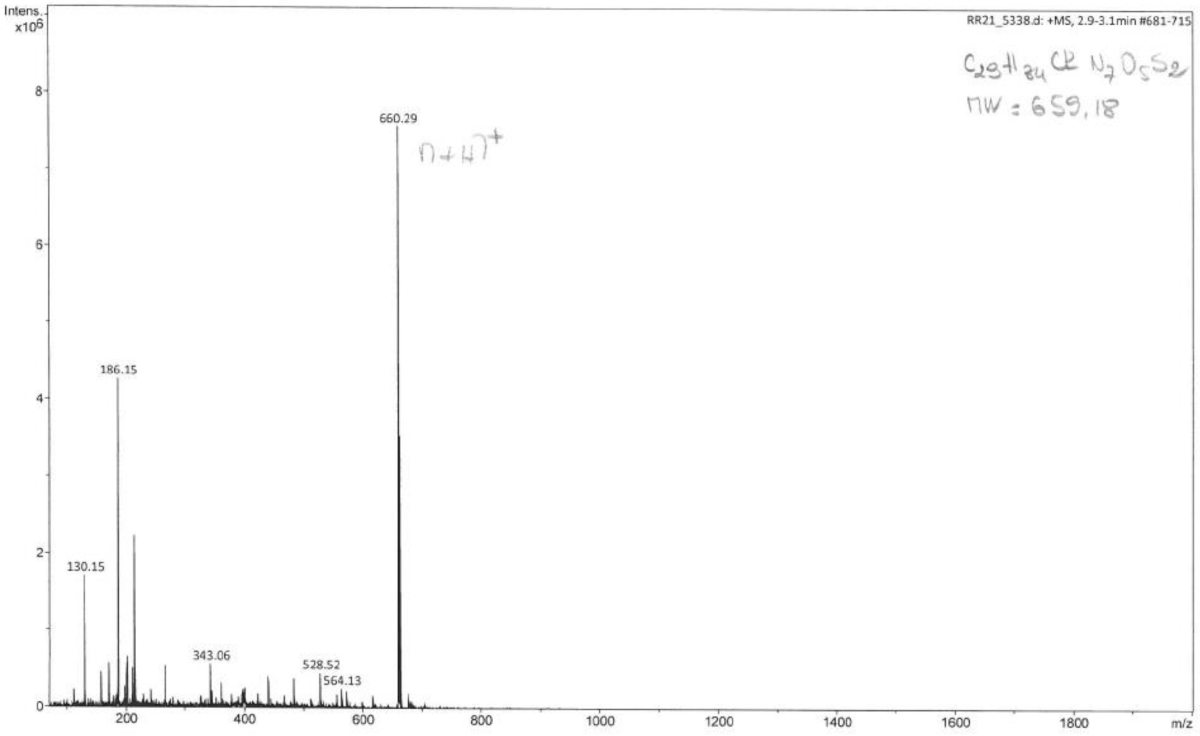

# *RR18 (MolPort-000-274-087)*

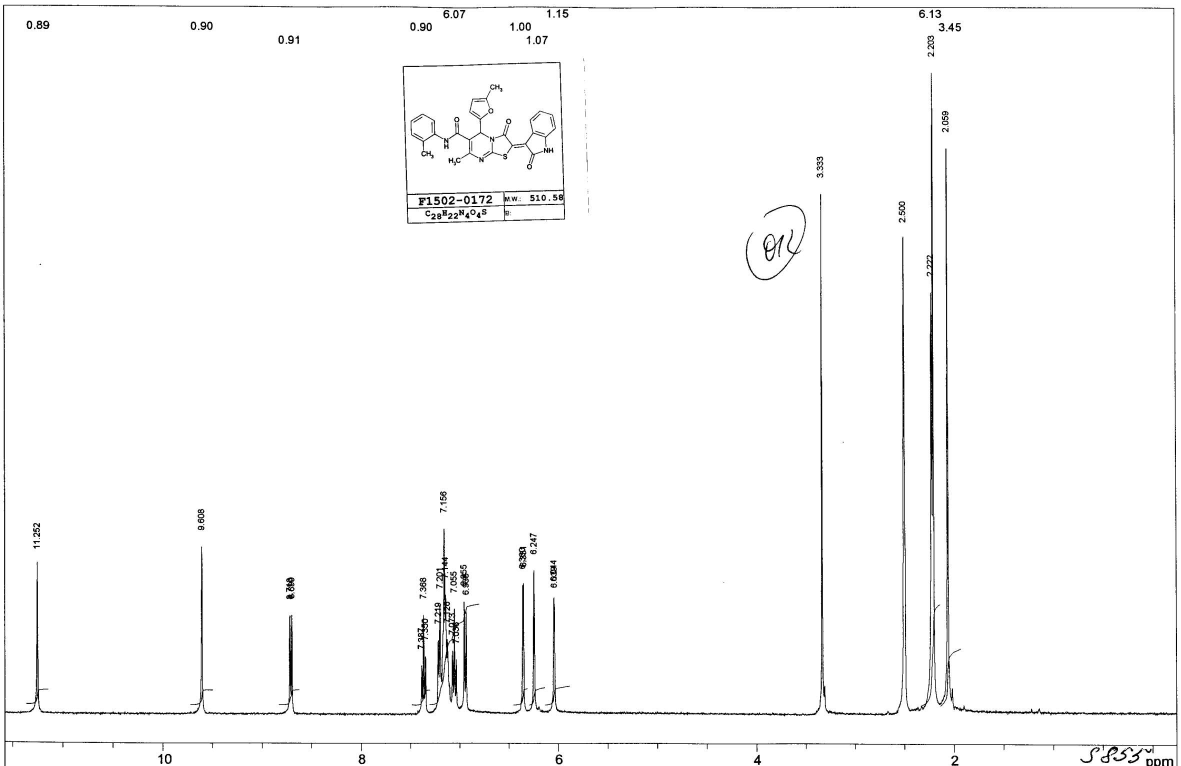


;f1502-0172 in DMSO;  USER:  DATE: Mar 19 2003

| | | | | | | |
|---|---|---|---|---|---|---|
| F1: 400.397 | F2: 400.396 | SW1: 8000 | | OF1: 2974.4 | | PTS1d: 16384 |
| EX: s2pul | | PW: 6.0 usec | PD: 1.0 sec | NA: 2 | LB: -0.8 | Nuts32 - f1502-0172.nmr |

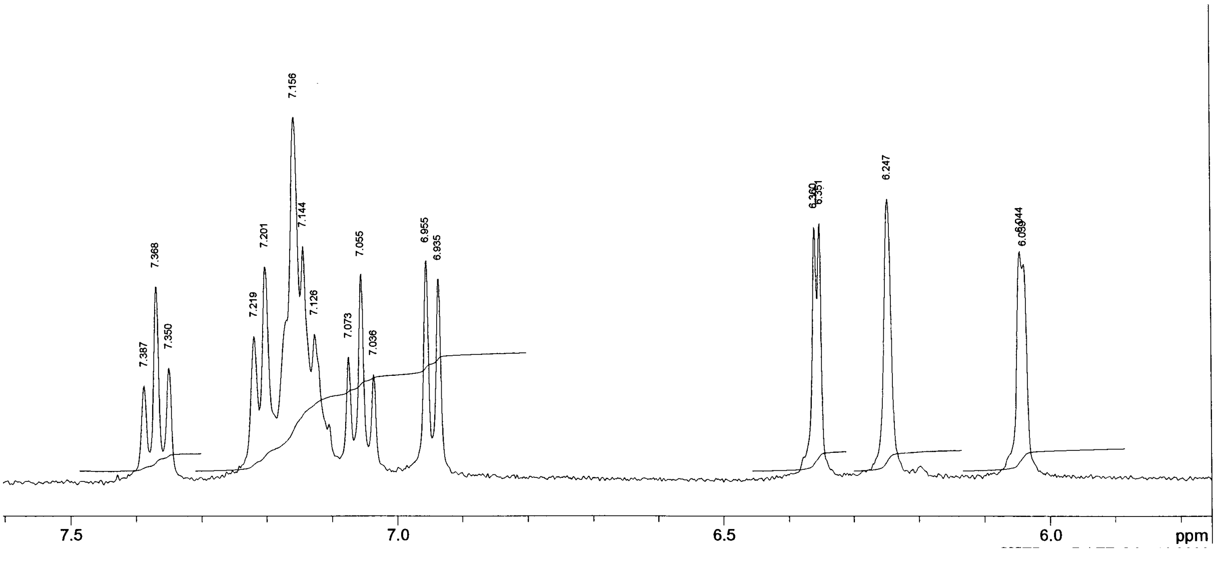

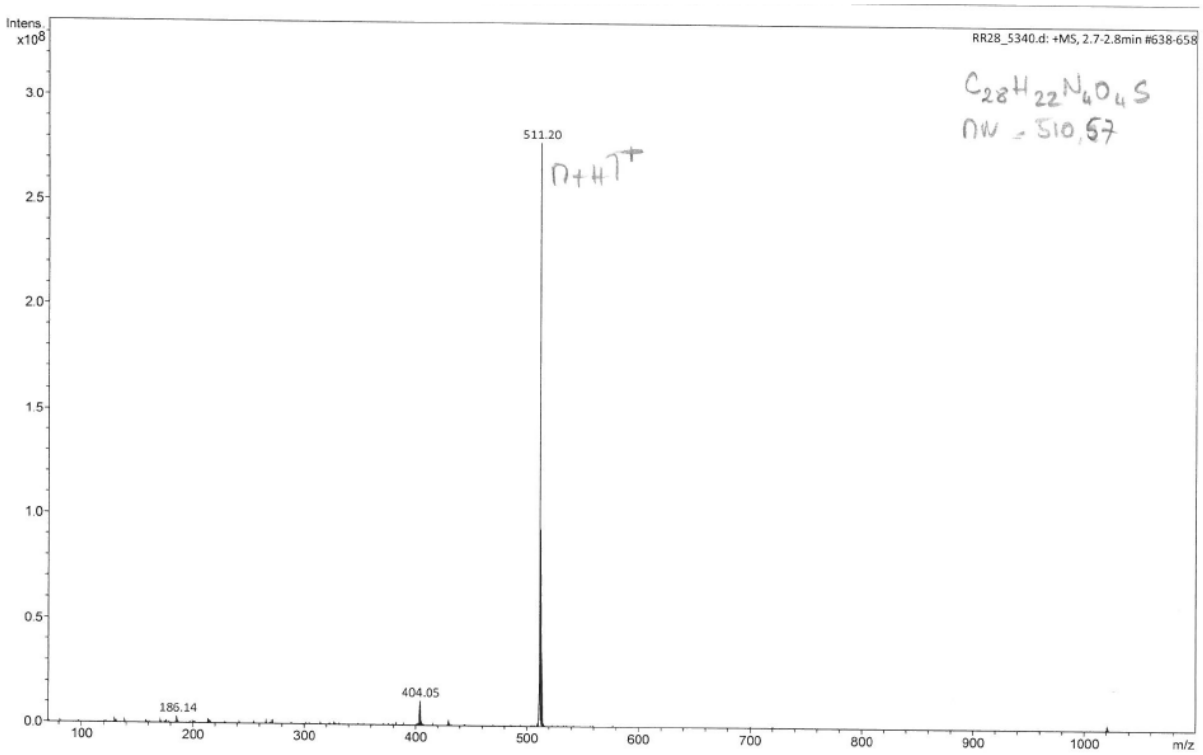
Intens.
x10^8
RR28_5340.d: +MS, 2.7-2.8min #638-658
C28H22N4O4S
MW = 510,57
511.20
M+H]+
404.05
186.14
m/z

## *RR20 (MolPort-000-117-304)*

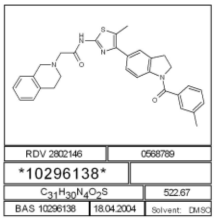


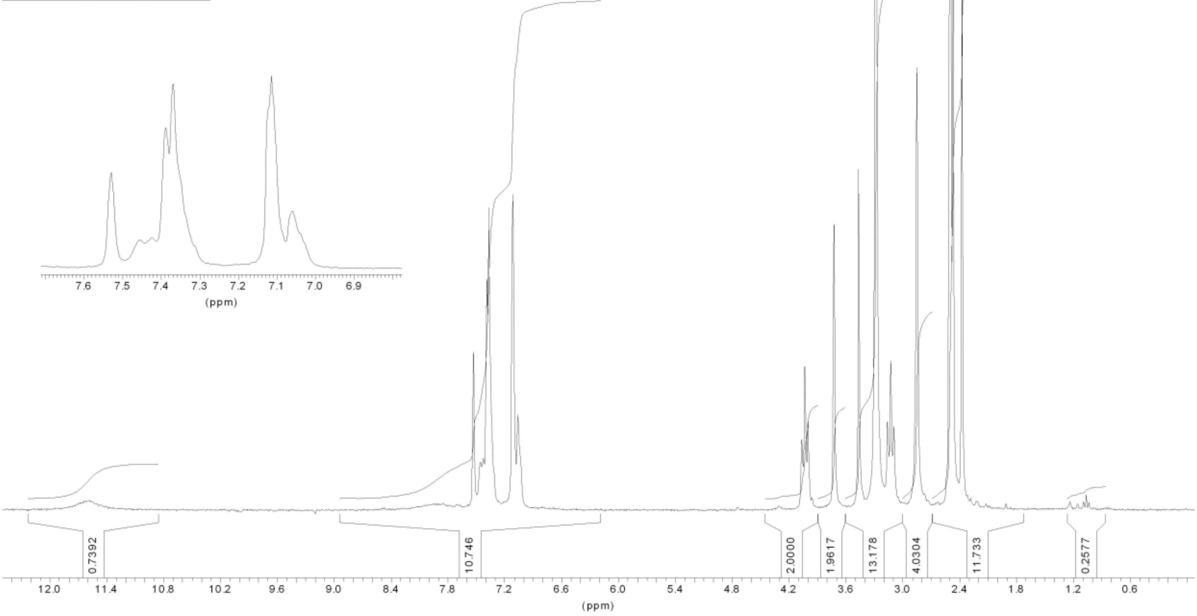


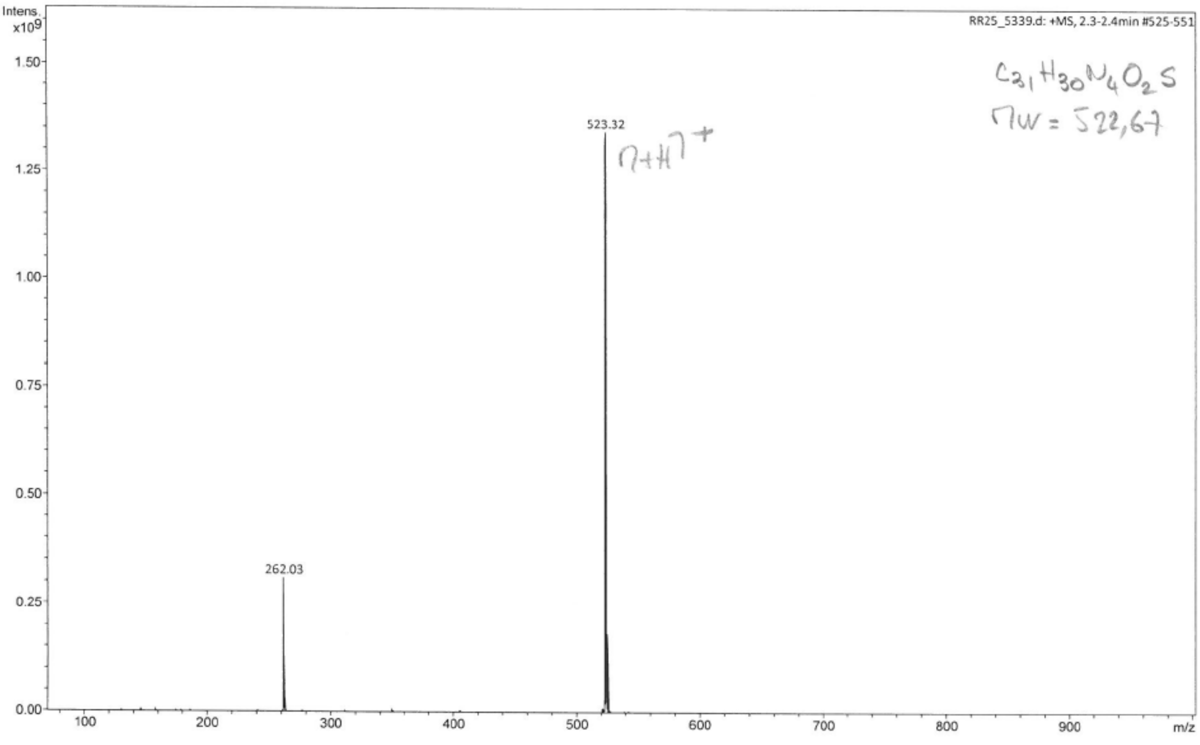

***RR21 (MolPort-000-250-727)***

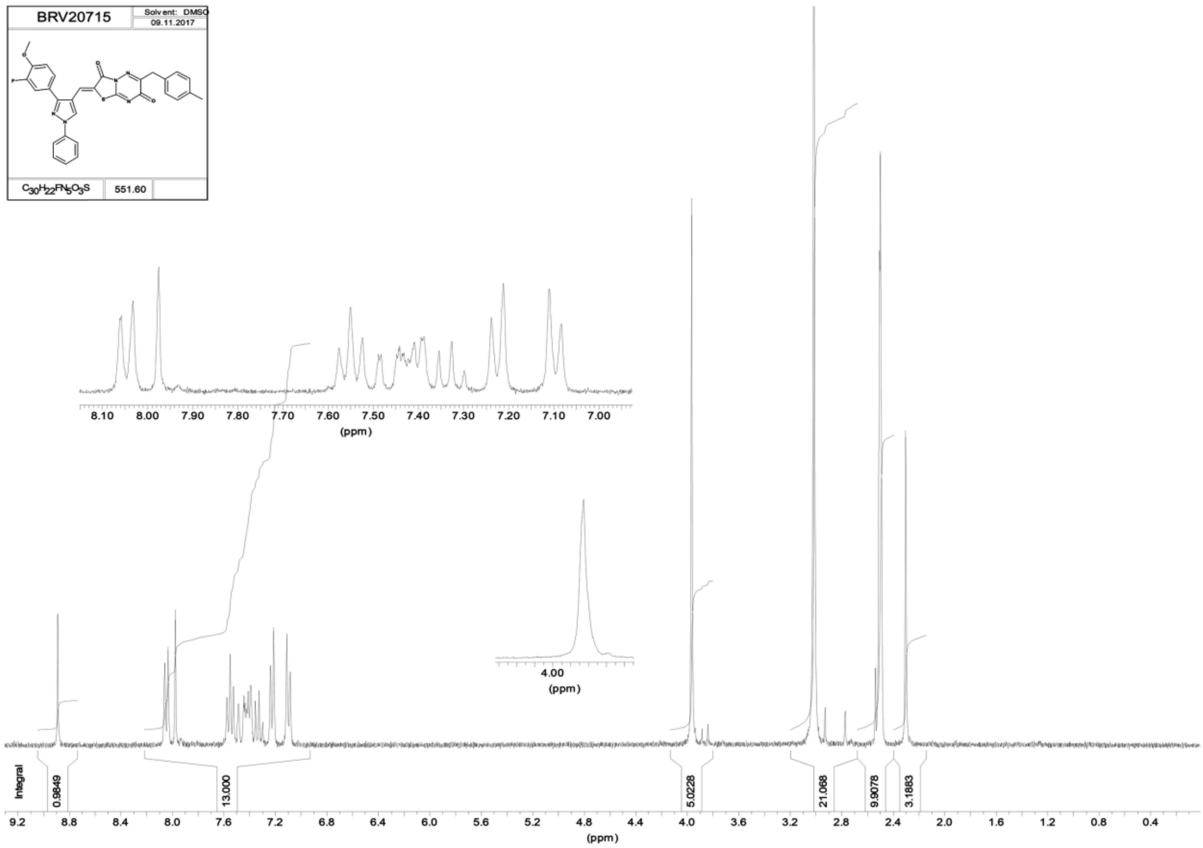


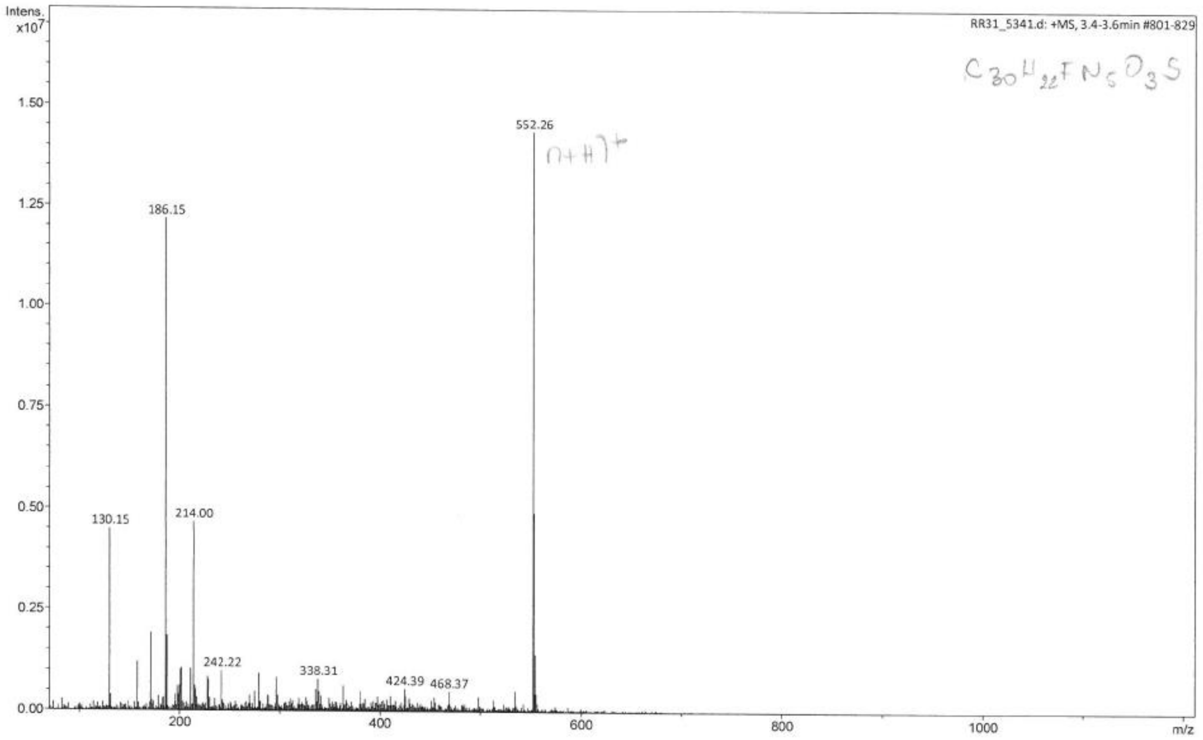

Supplement: S4 Fig — Comparison of the docking poses obtained with the five conformers for the most active compounds: (A) RR3, (B) RR4, (C) RR6, (D) RR9, (E) RR11, (F) RR16, (G) RR18 and (H) RR20. The binding pose of each compound is shown in stick representation with a color code according to the conformer used for docking (blue for C1, green for C2, yellow for C3, pink for C4 and orange for C5). (PDF) [file pcbi.1005943.s006.pdf]
